# Family relations of Moche elite burials on the North Coast of Peru (~500 CE): Analyses of the Señora de Cao and relatives

**DOI:** 10.1073/pnas.2416321121

**Published:** 2024-12-23

**Authors:** Jeffrey Quilter, Kelly Harkins, Régulo Fanco Jordan, Erik Marsh, Gabriel Prieto, John Verano, Steven LeBlanc, Nasreen Broomandkhoshbacht, John Krigbaum, Lars Fehren-Schmitz

**Affiliations:** ^a^Peabody Museum of Archaeology and Ethnology, Harvard University, Cambridge, MA 02138; ^b^UCSC Paleogenomics, Department of Anthropology, University of California, Santa Cruz, CA 95064; ^c^Régulo Franco Jordán, Parque Arqueológico Nacional de Machupicchu, Ministerio de Cultura, Cusco, Peru; ^d^CONICET, Laboratorio de Paleoecología Humana, Instituto Interdisciplinario de Ciencias Baásicas, Universidad Nacional de Cuyo, Mendoza M5500, Argentina; ^e^Department of Anthropology, University of Florida, Gainesville, FL 32611; ^f^Department of Anthropology, Tulane University, New Orleans, LA 70118; ^g^UCSC Genomics Institute, University of California, Santa Cruz, CA 95064

**Keywords:** Moche, ancient DNA, kinship, Peru, isotopes

## Abstract

The Moche archaeological culture flourished along Peru’s North Coast between the 4th and 10th centuries CE and was characterized by a complex social hierarchy dominated by political and religious elites. Previous archaeological evidence suggests kinship was a key factor in maintaining political authority within Moche society. To test this hypothesis, we applied archaeological, genetic, and isotopic methods to examine familial relationships between six individuals, including the prominent Señora de Cao (*~500 CE*), buried together in a pyramid-like, painted temple, Huaca Cao Viejo, in the Chicama Valley, Peru. Our findings reveal that all six individuals were biologically related, with varying degrees of kinship. The Señora de Cao was interred with a sacrificed juvenile, identified as a possible niece, and at least one, and potentially two siblings and a grandparent in separate tombs nearby. One of the male siblings was accompanied in death by his sacrificed son. Isotopic analysis indicates that while most individuals had diets rich in maize and animal protein and spent their childhoods in or near the Chicama Valley, the sacrificed juvenile accompanying the Señora had a distinct diet and geographic origin. These results demonstrate that Moche elites were interred with family members, including some raised far from their parental homes. This supports the hypothesis that kinship was central to transmitting status and authority. Moreover, sacrificing family members to accompany deceased elites underscores the significance of ritual sacrifice in reinforcing familial ties and linking the deceased to both ancestors and the divine.

## Significance

This study provides the first confirmation of familial relationships within an elite Moche burial group from Huaca Cao Viejo, offering new insights into Moche social organization, burial practices, and kinship-based politics. Using genomic and isotopic data from the burial group, including the prominent Señora de Cao and related individuals, we reconstructed a family tree spanning at least four generations and demonstrate that kinship played a central role in Moche elites’ political and ritual activities. The finding of sacrificed juveniles closely related to the main burials suggests a previously undocumented form of ritual sacrifice, underscoring the complexity of Moche ceremonial practices. This research contributes to understanding ancient Andean societies by illustrating the intersections between kinship, status, and ritual.

**Fig. 1.**
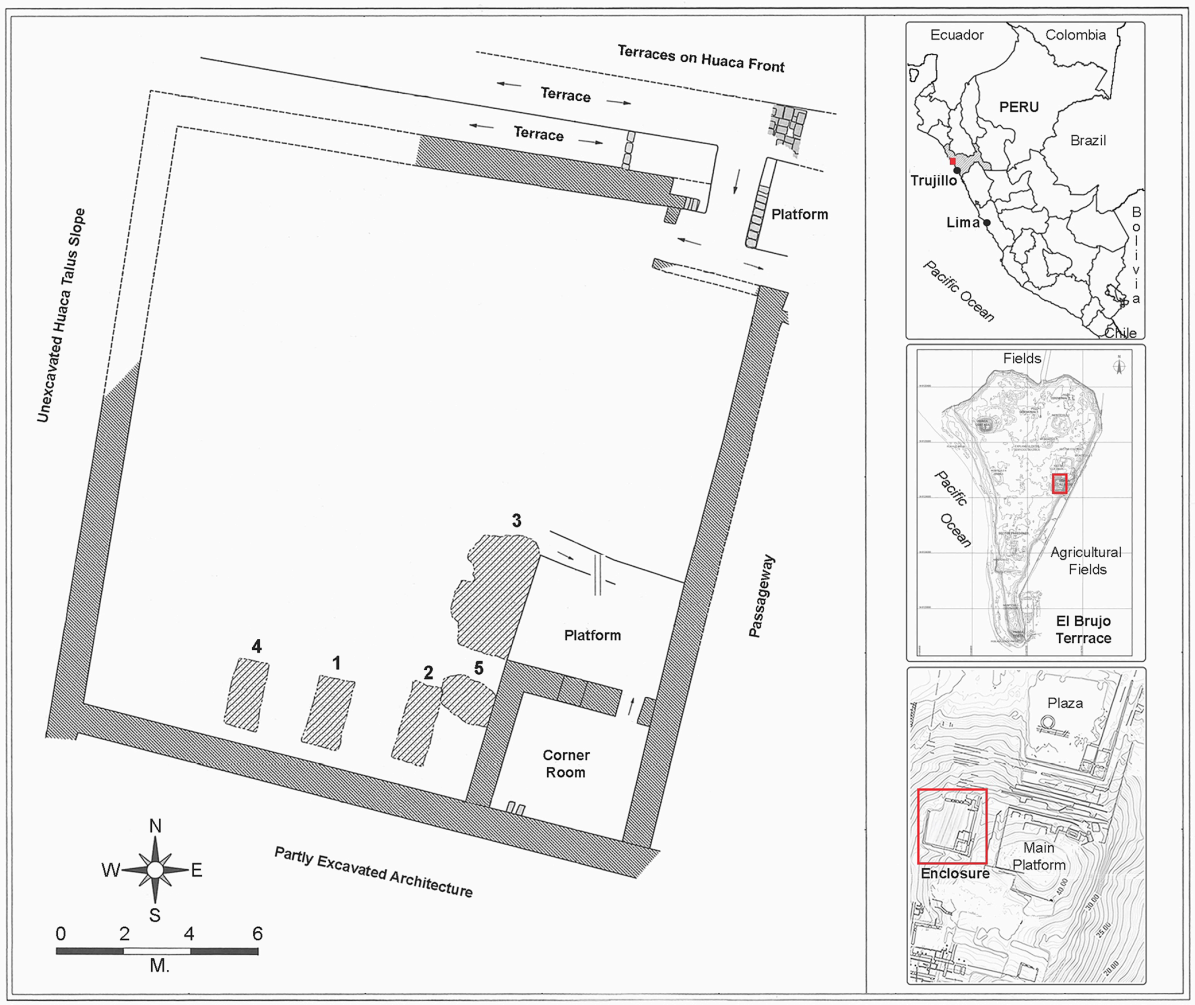
Map of the Señora Group Enclosure. *Right*, *Top*-to-*Bottom*: Locations of the El Brujo Archaeological Complex, the Huaca Cao Viejo, and the Enclosure in descending order *Left*: The enclosure with tombs numbered. Adobes and standing walls in gray and hatched. Arrows = ramp ascents.

The Moche archaeological culture occupied nine river valleys along the North Coast of Peru from *circa* 300 to 950 CE (Fig. 1) (1, 2), creating sophisticated urban complexes with monumental pyramid-like temples (*huacas*), irrigation networks, and elaborate major and minor arts (particularly metals and ceramics). A social hierarchy was capped by a political and religious elite who waged wars, impersonated deities in complex rituals, and elaborately buried their dead in large adobe *huacas* (1, 3, 4). The intricacies of intra- and intervalley politics remain to be fully elucidated but appear to have been complex, including influences, alliances, and pilgrimages (1, 5, 6). These and other issues are primarily investigated through archaeology, as the Moche did not leave any written sources.

Here, we present high-resolution genetic, archaeological, and isotopic data from an elite Moche burial group found in a walled enclosure at the Huaca Cao Viejo (Fig. 1), a 30 m-high adobe temple at the El Brujo archaeological complex in the Chicama Valley (7, 8), in order to determine their familial relations and life histories and to examine how kinship may have been linked to politics in Moche society. Discovered in 2005, the burial group, dating to circa *~500 CE (440 to 540, 95%)* (7, 9–11), consists of six individuals in four tombs made by removing bricks from the huaca floor.

## Description of the Archaeological Site and Tombs

Of the four tombs found in the enclosure at the Huaca Cao Viejo, three were found close to the southern wall (Fig. 1). Each contained an adult (20 to 30 y) male individual (Burials 1, 2, & 4 [B1, B2, B4]) in the extended, supine position. One individual (B1) was wrapped in seven layers of fine mantles, cloths, and cords and a reed mat. Under these, next to the body, was a wrapped object of white and red feathers, an elaborate vest ornamented with feathers, metal discs, and three painted ceramics. At the feet of the B1 burial was the flexed body of a juvenile individual (Burial 1-Sacrifice [B1s]) with a cord around his neck indicating death by strangulation, a known form of human sacrifice in the Moche culture (12).

East of B1, Burial 2 (B2), another adult male, was similarly buried: supine; head wrapped in cloth and with at least seven layers of textiles, including a tunic decorated with metal plaques and a reed mat as a final cover. B1 and B2 both exhibited partial preservation of soft tissues, while B1s was skeletonized. To the west of B1, another supine, fully skeletonized adult male, Burial 4 (B4), was the least well-preserved of the three internments. Only scant remains of textile fragments, reed mat, and cordage were preserved around his lower legs and feet. While the tombs of Burials 1 and 2 were at the same depth (1.2 m), the tomb for B4 was narrower and shallower (0.6 m) and likely more humid, increasing the deterioration of remains and perishable offerings in the tomb.

The fourth tomb was separate from the three others in a row, close to the enclosure wall. It was situated next to the small platform of an elaborately painted room in the SE corner of the enclosure. At a depth of 3 m, the well-preserved remains of a female adult, commonly referred to as the Señora de Cao (Burial 3 [B3]), had been placed, wrapped in more than 20 layers of textiles and offerings including ceremonial spear-throwers, two large ceremonial clubs, gold crowns and nose ornaments, and various other items. The male-gendered weaponry and nose ornaments and the female-gendered items suggest that the Señora de Cao had a very high status (1, 10, 11, 13). Next to the outer layer of the Señora’s bundle lay a sacrificed juvenile female (B3s) with a rope around her neck (12).

The Señora burial is significant as the best-preserved elite burial found in Peru to date, and using the archaeological dictum that the amount of energy expenditure in tomb construction and offerings indicates relative social rank, the Señora had the highest status among the other burials, although all the main burials were of high status given that they were interred in the temple. The less elaborate grave goods, poorly preserved skeleton, and the shallower tomb of B4 suggest that this individual may have been initially buried elsewhere and then moved to the patio to accompany the others, a known Moche practice.

All the individuals buried in the enclosure were examined for evidence of skeletal and dental pathological conditions. Causes of death were identified in only two individuals, the juveniles strangled with plant fiber ropes (B1s, B3s). Skeletal and dental pathologies observed with the individuals buried in the patio were minimal and included only minor conditions common in prehistoric populations of the north coast of Peru. The exception is B1, which shows remodeled subperiosteal inflammation of the shafts of multiple leg and arm bones, indicating some general inflammatory process that was healed at the time of death (14).

A pottery vessel found above the level of the Señora’s tomb with its lip protruding above the floor served to receive offerings (9). In addition, the remains of burnt offerings, nearby, on the floor indicated that postburial rituals were conducted to honor the deceased. A date for a ritual fire on the floor above the sealed tombs provides a *terminus ante quem* (*~660 CE*; Dataset S1) for the entire burial group and suggests that the memory of the dead was maintained for a considerable length of time after death. Eventually, however, the enclosure was filled with a thick layer of adobe bricks by later Moche, who continued to increase the size of the Huaca de Cao with new constructions.

## Results

We sequenced low-coverage genomes for all six individuals (0.01 to 1.2x coverage) from single-stranded DNA sequencing libraries, largely exhausting the preserved unique endogenous molecules in the libraries (Dataset S2). DNA preservation was a major limitation to our sequencing efforts (*SI Appendix*, Fig. S1); however, damage at the terminal ends of the sequencing reads, and low estimated contamination rates indicate that the data obtained are authentic (Dataset S2*A*). The genomic data confirm morphological sex estimates for all four adult individuals and allow us to determine the biological sex of the two sacrificed subadults, with B1s as male and B3s as female (Dataset S2*A*). In addition to the genomic analyses, radiocarbon dates for all six individuals were performed (Dataset S1), and multiple isotope ratios were generated from strontium, lead, carbon, oxygen, and nitrogen isotope data providing information on their origins, mobility, and diets (Table 1 and Dataset S3).

**Table 1.** Light and heavy isotope ratios for Huaca Cao Viejo individuals sampled

				δ^13^C_col_^1^	δ^15^N_col_^*^	δ^13^C_en_	δ^18^O_en_	Δ^13^C_en-col_						
Ind	Age (yrs)	Sex	Tooth	(‰, VPDB)	(‰, AIR)	(‰, VPDB)	(‰, VPDB)	(‰, VPDB)	% C_4_^†^	% Marine^‡^	^87^Sr/^86^Sr^§^	^208^Pb/^204^Pb^§^	^207^Pb/^204^Pb^§^	^206^Pb/^204^Pb^§^
B1	A (25 to 30)	M	M3	−11.1	10.7	−6.4	−4.2	4.7	70.8	50.8	0.70882	38.724	15.655	18.847
B1s	SA (12 to 13)	M	M1	−11.3	8.9	−6.7	−4.4	4.6	68.8	37.4	0.70697	38.713	15.632	18.851
B2	A (20 to 25)	M	rib	−9.3	11.9	N/A	N/A	N/A	N/A	70.7	N/A	N/A	N/A	N/A
B3	A (25 to 30)	F	M1 root	−12.0	10.4	N/A	N/A	N/A	N/A	44.7	0.70882	38.832	15.662	18.961
B3s	SA (12 to 15)	F	C	−13.4	8.6	−11.4	−6.1	2.0	35.0	30.3	0.70919	39.411	15.762	19.911
B4	A (20 to 25)	M	M2	−9.9	11.5	−5.9	−4.2	3.9	74.2	64.5	0.70878	38.869	15.674	18.992

^*^Data reported in AMS results from Keck Carbon Cycle AMS Facility, UC Irvine (AMS #235036 - 235040; 270812; Dataset S3).

^†^% C_4_ was determined using −26‰ as endpoint for 100% C_3_, −12‰ for 100% C_4_, and 9.7‰ δ^13^C_en-diet_ spacing in the formula %C_4_ = (−26−(δ^13^C_en_ − 9.7)/−16)*100 (adapted from Somerville et al., 2013).

^‡^% Marine parameters follow Richards (2020:138, Fig. 6.1) with reasonable guesstimates for bone collagen: δ^13^C values: marine protein [−11.9‰ (±2.2‰)], terrestrial mammals [−16.6‰ (±1.3‰)], C_3_ plants [−25.7‰ (±2.1‰)], C_4_ plants [−11.3‰ (±1.4‰)]; δ^15^N values: marine protein [+12.7‰(±2.7‰)], terrestrial mammals [+8.0‰ (±1.2‰)], C_3_ plants [+5.3‰ (±3.2‰)], C_4_ plants [+7.3‰ (±3.0‰)](*SI Appendix*).

^§^Error for strontium and lead ratios included in Dataset S3.

### Genetic Affinities and Ancestry.

**Fig. 2.**
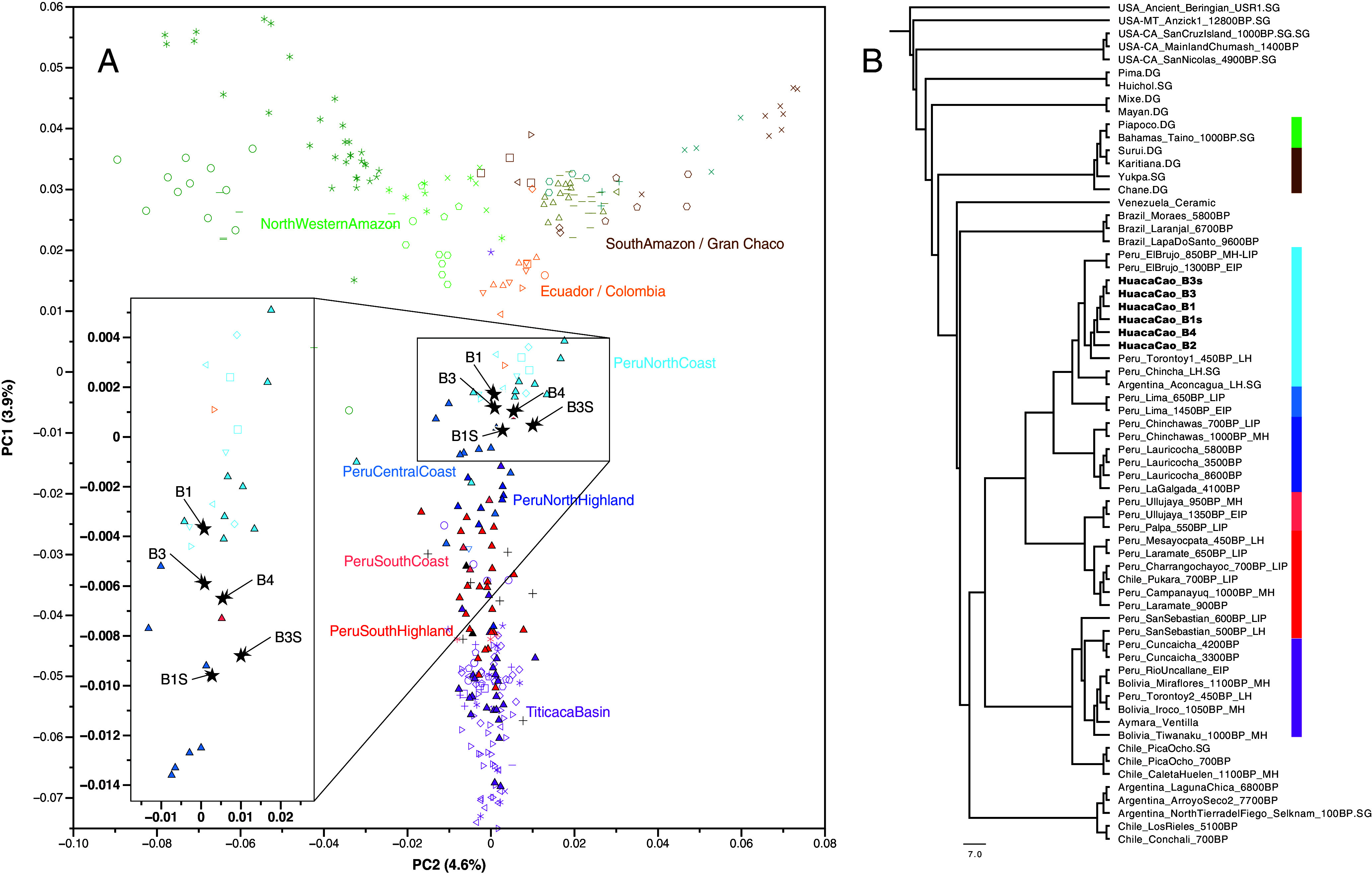
Genetic Affinity of the individuals from Huaca Cao. (*A*) PCA plot projecting previously published ancient individuals and the ancient individuals studied here onto principal components calculated from present-day Native American populations. The individuals buried in the patio of the Huaca Cao are depicted by black stars (extended version of PCA including all population labels in *SI Appendix*, Fig. S2). (*B*) Neighbor-joining tree based on inverted outgroup-f3 statistics [1/f3(Mbuti; Group1, Group2)] using the HO_OMNI SNP set showing the genetic affinity between the Huaca Cao individuals and other South American groups. The USA_Ancient_Beringian.SG individual (USA-AK_USR1_Beringian_1140BP.SG) was used as an outgroup for the tree. The color bars refer to the population coloring scheme used in the PCA plot. The bars only appear for populations overlapping in both analyses: the PCA and the F3-NJ Tree.

To investigate their genetic ancestry, we merged the six genomes with published ancient (n = 96) and modern-day genomes (n = 273) from Central- and South America (15–18). We projected 5 of the 6 individuals (B2 had insufficient data) together with previously published ancient individuals onto a set of modern South and Central American individuals using principal components analysis (PCA) (Fig. 2*A* and *SI Appendix*, Fig. S2). All 5 individuals fall within the PeruNorthCoast cline defined by Nakatsuka et al. (17). Additionally, outgroup-f3 statistics of the form f3 (Mbuti; X,Y), where X and Y are any ancient or modern-day population from the Americas or one of the Huaca Cao patio individuals, show that the latter share most genetic drift with other individuals of North Coast ancestry (Fig. 2*B*). However, f-statistics of the type f4 (Mbuti, X; HCV, Peru_ElBrujo_EIP), where X is any population in the dataset, HCV is one of the enclosure individuals, and Peru_ElBrujo_EIP are genomes from contemporary Moche commoners buried in the surrounding El Brujo complex (17), indicate that the elite individuals do not form a strict clade with the Moche Period commoners but that the latter share more alleles with groups from the South Central Peruvian Coast and Highlands, relative to the former, for example, with Peru_Laramate_900BP (f4= 0.004, Z = 5.8) or Peru_Ullujaya_1350BP_EIP (f4=0.003, Z = 4.1; (Dataset S2*B*).

### Isotopic Perspectives on Origins/Residence.

As a complement to ancestry analyses, we conducted isotopic analyses of individual tooth enamel (B1, B1s, B3s, B4) and tooth root (B3, Señora de Cao) following established procedures for light isotopes (19) and for heavy isotopes including radiogenic strontium (^87^Sr/^86^Sr) (20, 21), and lead (^20n^Pb/^204^Pb) (22, 23). Radiogenic isotopes complement stable isotope ratios of carbon (δ^13^C) and oxygen (δ^18^O) derived from the same sample of tooth enamel, particularly when faced with issues of equifinality for one or more isotopic systems.

**Fig. 3.**
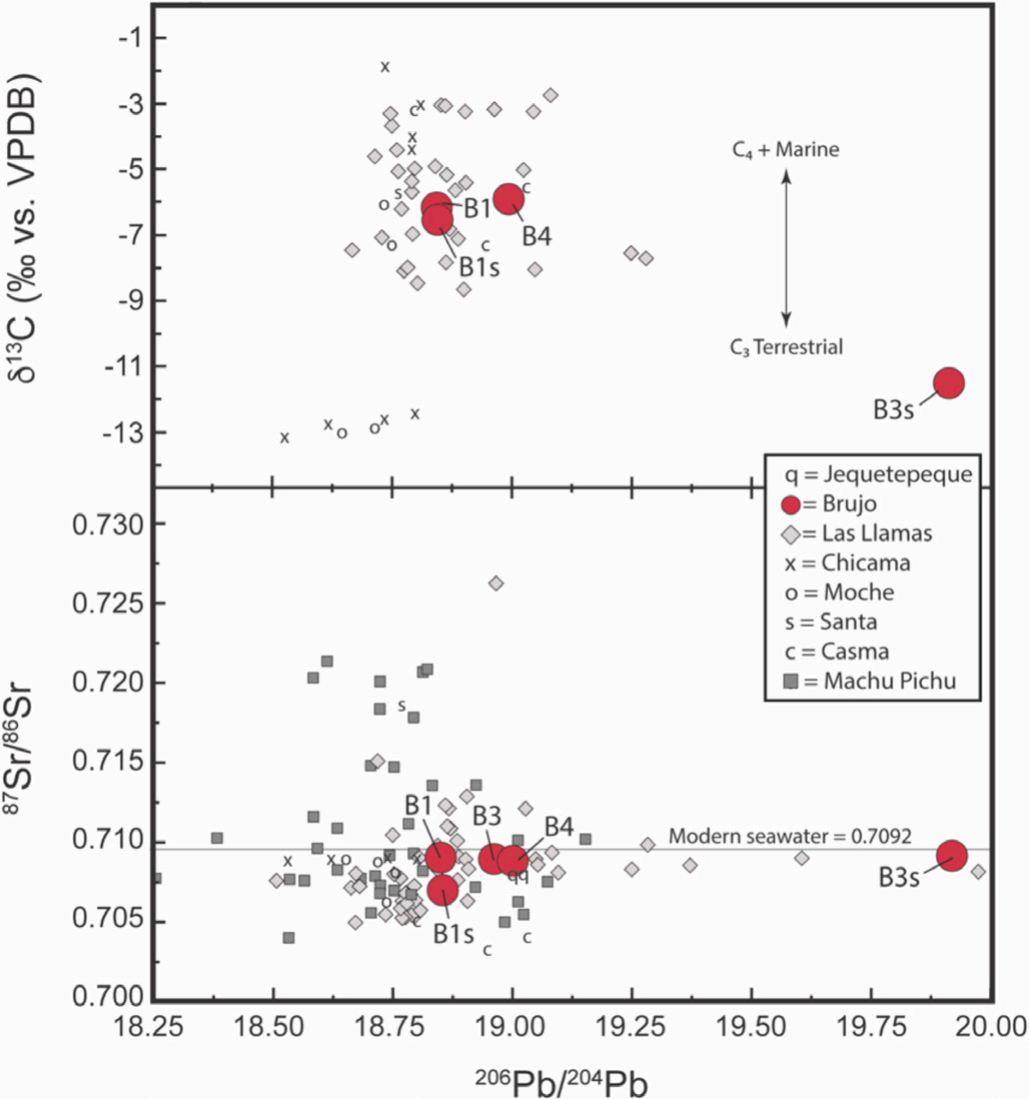
Select strontium (^87^Sr/^86^Sr) and carbon (δ^13^C) isotope ratios vs. lead (^206^Pb/^204^Pb) for Huaca Cao individual tooth enamel and comparative site samples (*SI Appendix*, Table S4), including nearby HLL (*SI Appendix*, Table S5). Señora de Cao (B3) includes root dentin assayed only for strontium and lead.

The geology of northern Peru is punctuated by the Andes running parallel to the coast. Valleys cutting through the mountains expose Quaternary sediments which include Tertiary volcanics. Pb provinces (24, 25) for central and southern Peru may extend into northern Peru, but are not ground tested. The Pb provinces, that broadly discriminate Pb isotope groupings, include Province I (coastal), Province II (interior to coast), and Province III (further inland and Highlands) (*SI Appendix*, Fig. S6 and Dataset S3). To appreciate isotopic diversity observed with the patio burials, opportunistic sampling of prehistoric remains recovered from select valleys was integrated into plots demonstrating where the patio burials fall in terms of broad similarity (Fig. 3 and Dataset S3). Huanchaquito–Las Llamas (HLL), a site dating to 1400 to 1450 CE, less than 20 km south of El Brujo, is isotopically the most varied (Fig. 3 and Dataset S3), demonstrating the potential range of individuals in the region, and published data from Machu Picchu, to the south, is included for comparison of Pb and Sr (23, 26).

Isotopic results (Table 1) show broadly consistent trends of residence and diet for four of the five individuals (B1, B1s, B3, B4). For strontium (Table 1), B1 and B3 have nearly identical values (0.70882) and are broadly comparable to B4 (0.70878). The two sacrificed individuals are different: B1s, with a lower value (0.70697), is more consistent with local predicted values in the Chicama Valley (20), whereas B3s, with a higher value (0.70919), is more consistent with either coastal or more interior (likely highlands) values that stretch the length of northern Peru (20, 21). It should be noted that the signal for B1s is derived from a first molar and thus more likely its value reflects its mother’s residence. With respect to Pb, Senora de Cao (B3) and B4 share similar Pb values as do B1 and B1s; however, B3s is an extreme outlier (Fig. 3 and *SI Appendix*, Fig. S6). Both B1s and B3s also have lower nitrogen (δ^15^N) values compared to the other burials, and B3s has the lowest observed δ^13^C values for both sampled tooth enamel (and tooth dentin) and relatively low δ^18^O, which would support “nonlocal” (inland) residence (Table 1). Trace element and rare earth element concentration data (27) for Huaca Cao individual samples suggest minimal alteration (*SI Appendix*, Fig. S7).

### Dietary Observations.

Most of the Huaca Cao burials show minor dietary differences, with the exception of B3s. Estimates of dietary % C_4_, often associated with the consumption of maize, suggest that B1, B1s, and B4 exhibit about 70% C_4_ compared to outlier B3s, with around 35% (Table 1). The highest estimated marine diet component, based on the δ^15^N values, was 65 to 70% (±21%) for B2 and B4, followed by 45 to 50% (±23.5%) for B1 and B3 (Table 1 and *SI Appendix*, Table S3). The δ^15^N values of both sacrificed individuals were ^15^N depleted (lower) relative to others. Here, again, B3s exhibit the lowest values, with an estimated marine diet component of about 30% (±18%) (Table 1 and Dataset S3).

### Biological Relatedness and Kinship.

We utilized five computational tools to infer pairwise biological relatedness between the six Huaca Cao Viejo individuals from the genomic data (28–32) (*SI Appendix* and Dataset S2). Collectively, the results indicate that the six individuals share a close degree of biological relatedness (Dataset S2 *C*–*F*): B1 and the Señora (B3), and B1 and B1s are 1st degree relatives. Two of the methods, KIN and lcmlKIN, aim to distinguish between two types of 1st degree relatedness: sibling and parent–offspring (29, 31). These results indicate that the Señora and B1 are siblings (KIN: log likelihood = 12.04; lcmlKIN: r/k0 = 1.7239), while B1 and B1S (KIN: log likelihood = 29.22; lcmlKIN: r/k0 = 0.8787) are parent–offspring (Dataset S2*C* and *SI Appendix*, Fig. S4). While the number of SNPs overlapping between B2 and all other individuals is minimal (≤ 2,000 SNPs, Dataset S2 *C*–*F*), all applied methods consistently determine B2 and B1 to be 1st degree relatives (Dataset S2*C* and *SI Appendix*), with KIN suggesting that they are siblings (Log Likelihood = 4.65; Dataset S2*C*); however, the r/k0 ratio (=6.5) determined with lcmlKIN suggests that they could be parent–offspring. B1, B1s, B2, and the Señora (B3) all share the same mitochondrial DNA haplogroup D lineage (subsequently named mtD-1, to distinguish from the different haplogroup D lineage observed with B3s, here mtD-2), suggesting maternal relatedness. We further observed 2nd degree relatedness between the pairs B1–B4, B3–B4, B3–B1s, while B4 and B1s are potential 3rd degree relatives (Dataset S2*C*).

While the low coverage obtained for B3s severely limits our ability to determine the exact degree of relatedness to most of the others, all methods consistently indicate that B3 and B3s, the Señora and accompanying sacrifice, are 2nd degree relatives (Dataset S2). We also computed Runs of Homozygosity (ROH) for the datatwo individuals with the highest coverage (B1 & B4). The presence of higher frequencies of long ROH (>15 cM) in some genomes typically indicates an increased degree of parental relatedness (33). The level of long ROH observed in the genome of B4 (sum_ROH > 20 cM = 54.45; *SI Appendix*, Fig. S5) suggests that this individual’s parents must have been 5th degree relatives (e.g., second cousins), while the parents of B1 were not related (> 3rd cousin) (*SI Appendix*, Fig. S4).

### Family Tree Reconstruction and Dating.

**Fig. 4.**
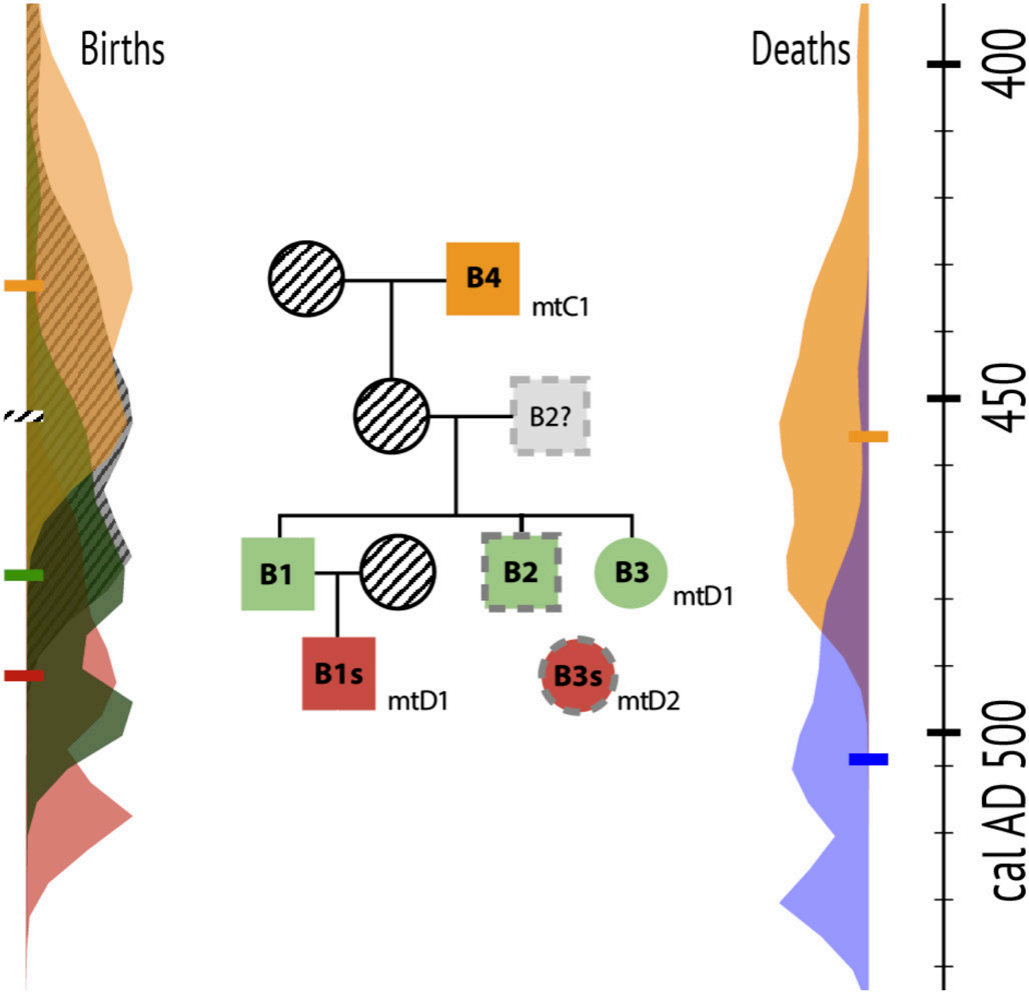
Reconstructed family tree based on the genomic and contextual data aligned with results from the Bayesian chronological model. The family tree was drawn with Cranefoot and edited. The observed mitochondrial lineage for each individual is found below the symbol of each individual (mtD-1 and mtD-2 represent different lineages, both belonging to mtHaplogroup D). Dotted outlines indicate unresolved positions in the family tree. Unsampled individuals have diagonal hatching. The right side shows chronological probability distributions and medians (short horizontal bars) for birth dates, with matching colors in the family tree. The left side shows probability distributions for death dates. The blue distribution is for the combined event for Tombs 1 to 3, including grave goods and the deaths of all dated individuals except B4. All details on the Bayesian Models can be found in *SI Appendix 1: Radiocarbon Date Calibration & Modeling*

We reconstructed a family tree spanning at least four generations for the Huaca Cao Viejo individuals (Fig. 4 and Dataset S2*C*), drawing on several lines of evidence: The consensus kinship estimates based on the autosomal data; the mitochondrial haplotypes to indicate maternal kinship; the individual age at death, excluding subadult individuals as potential parents; the radiocarbon dates obtained from skeletal remains of the individuals, and other information deriving from the archaeological context (*SI Appendix*).

To obtain a better understanding of the chronological relationship of the individuals, we built a Bayesian model that includes the five newly obtained dates on human tissue and eight dates on materials from Tombs 1 to 4 (Dataset S1). Treated as a single depositional event, the model suggests that B1, B1s, B2, B3, and B3s died and were entombed *~500 CE (440 to 540, 95%)*, with a good agreement index (88%). Individual B4 died *~460 CE (390 to 500, 95%)*, about 40 y before the others. The model cross-references generational gaps and family relationships identified by genomic data (Fig. 4), providing crucial temporal constraints that significantly improved otherwise notably imprecise calibrations. The model uses birth and death dates for each individual, estimated from ages of dated tissue formation, ages at death, and percentages of marine protein in their diets (Dataset S1). There are highly variable ΔR estimates for this region due to variable upwelling, so we let this parameter vary freely (34). Based on individual diet estimates and the other constraints, the model estimates that the local ΔR was *−270 ± 72 y* at the time.

The combined data indicate that the earliest dated individual of the burial group, B4, was the grandparent of the two putative siblings B1 and the Señora (B3), the highest-status burials of the group. The remains of B4 were most likely reburied in the patio, a few generations after his death, in the patio when the main burials were interred. The genetic relationship between B1 and B2 suggests that the latter could have been either the sibling or parent of B1 and the Señora. There is not enough overlap in the genetic data between B2 and the other burials to securely determine which alternative is more likely. B2’s birth date is consistently modeled as around *~AD 480*, which aligns with the estimated birth dates of both B1 and B3, and makes it much more likely that B2 is B3's sibling (Fig. 4 and Dataset S1).

We find robust support that both sacrificed juveniles (B1s, B3s) were relatives of the main burials with whom they were interred, B1 and the Señora, respectively. In the parent–child case of B1 and B1s (both related to the Señora), there are no indications that the tomb had been reopened and the placement of the individuals in relation to each other suggests simultaneous burial. In the case of the young female (B3s) sacrificed to accompany the Señora de Cao, there is some indication that the individual could be a 2nd degree (niece or grandchild) relative to the Señora. The age-at-death of B3s was 12 to 15 y and morphological analyses suggest that the Señora was no older than 30 y at death (14). Both individuals were buried at the same time. Thus, it is highly unlikely that the Señora would have had a granddaughter of that age at the time of her death, ruling out that relationship. While the genetic data do not allow us to determine the absolute placement of B3s on the family tree, our models indicate that this individual was probably born within a few years of B1s (Fig. 4 and Dataset S1). The age-at-death of B3s and the 2nd-degree relationship to B3 suggests that the individual was B2's child, the putative sibling of B1 and B3, or another unknown sibling.

## Discussion

Five other Moche elite burial groups have been excavated and reported since the late 1980s^*^. Many of these contained multiple individuals inferred to have been family members based on the archaeological record (3, 4, 35). Our study of the Señora Group Burials confirms that this was so at Huaca Cao Viejo, the first time that this has been scientifically demonstrated. Similarly, Moche art depicts females in special roles as goddesses and priestesses (36), the burials of priestesses at San José de Moro (*ca.* CE 700) attest to women’s important ceremonial roles (37), and early Spanish accounts from the 16th century of the peoples of the North Coast of Peru reported that women held positions of great authority (38). The discovery of the Señora de Cao demonstrated that women indeed held high rank in Moche times, at least two centuries earlier than San José de Moro, and a thousand years before Spanish reports of politically important women.

The Señora was buried with qualitatively richer and quantitatively more burial goods than any of the other elites in the Huaca Cao enclosure. The most important offering was the sacrifice of a juvenile, an honor also bestowed on the second-richest burial, the Señora’s brother, Burial 1. Remarkably, the other adult males, B2 and B4, were buried with few grave goods, yet they were closely related to the Señora de Cao. While burial in a huaca alone was a mark of high rank, the relatively few grave goods in those tombs suggest that notions of wealth and status were complicated in Moche culture. The lack of regalia or offerings suggests that proximity to the Señora in death was prioritized over other factors for the siblings and grandfather. Future studies that identify genetic relations between Moche elites buried in proximity should help to clarify such issues.

Of particular significance are the close genetic relationships observed between the sacrificed juveniles and the other burials. While the practice of human sacrifice and even sacrificial mass events have been well established in the archaeological record for the Moche and other Peruvian groups (26, 39, 40), no similar closely related sacrifices have previously been considered for the Moche (41, 42). A report from the Colonial Period provides an example of a father who sacrificed a daughter in Inca times (43), but in a different context a thousand years later and a thousand kilometers distant from the Moche of the North Coast of Peru.

Most burials in our study exhibited only minor health-related problems. Except for one individual, they all had diets that included maize and marine-derived proteins, and all likely were born and raised in or near the Chicama Valley or close to it. The sacrificed juvenile female (B3s) who accompanied the Señora (B3), stands apart both in terms of diet and geographic origins, although the individual was a close relative of the Señora (as were the high-status man (B1) and his sacrificed son (B1s). The upbringing of the sacrificed individual, likely in the highlands well beyond the Chicama Valley, underscores the importance of long-distance connections in ancient Peru. That a close relative, potentially a niece, was raised afar only to be sacrificed alongside their relative offers many possibilities for consideration and further supports the other evidence that both local and interregional Moche politics had a strong, likely dominant, kinship-based component. At present, we cannot determine whether the practice of sacrificing juvenile family members or other close relatives was part of the sociocultural norm, or due to the kinds of court intrigues commonly known for many societies through time and space.

The Moche were only one of scores of documented ancient societies in the Andes. Vast ruins of temples, cities, and shrines across the landscape and the rich materials record of the past bear witness to complex relations between peoples and societies over hundreds of kilometers. New ways of investigating the past hold great promise to enrich our understanding of them.

## Materials and Methods

### Sampling.

The remains of all individuals investigated here are curated at the Museo de Cao, El Brujo Archaeological Complex, Magdalena de Cao district, Department La Libertad, Perú. The remains of the individual known as Señora de Cao (B3) are part of a permanent exhibition at the museum. A team of Peruvian researchers associated with the museum, members of the UCSC Paleogenomics Labs (UC-PGL), and JQ conducted sampling in 2017. Following strict precaution measures for acquiring ancient DNA (aDNA) samples (44), teeth and metatarsal bone elements were extracted surgically to cut the smallest pieces of root and bone. The teeth and bone elements sampled were subsequently reinserted into their original context to leave no visible signs of the sampling, however, small chips of tooth enamel ( around 50 mg) were taken for isotopic analyses.

### Ancient DNA Laboratory Work.

The samples from the six individuals were processed at the UC-PGL clean room facilities at the University of California, Santa Cruz, following strict precautions for contamination prevention as described previously (17, 44). Fractions of each sample were shipped to the University of Florida (UF) for isotopic analyses and to the UC Irvine KECK AMS facilities for radiocarbon dating. After several methodological adaptations necessary because of the poor DNA preservation (*SI Appendix*), the data presented here were produced from DNA extracts generated using a silica-column-based protocol optimized for the recovery of small ancient DNA molecules (45), adding a 0.2% bleach predigestion (46), using 30 mg of sample. Subsequently, we built double-indexed single-stranded DNA sequencing libraries from the extracts (47). The libraries for each individual were sequenced on several lanes of a HiSeq4000 (Illumina) sequencer for 2 × 150 cycles at Fulgent Genetics (Temple City, CA). All extractions, library batches, and PCR amplifications were accompanied by negative controls.

### Sequencing Read Processing, Chromosomal Sex Determination, and Screening and DNA Authenticity.

After demultiplexing, resulting sequencing reads were processed using the in-house computational pipeline developed for aDNA described previously (48), available at (https://github.com/mjobin/batpipe). This pipeline merges paired-end reads (default parameters), maps sequencing reads against a user-specified reference genome, removes duplicate reads, and estimates quality traits. All shotgun-sequenced reads went into mapping with BWA (v0.6.1) (49) against the human genome reference GRCh37/hg19. Mitochondrial DNA (mtDNA) reads were mapped against the human mtDNA reference rCRS (50).

Chromosomal sex was determined by evaluating the ratio (Ry) (51). In addition, we employed a X-chromosomal normalization rate (Rx) approach that compares the Rx ratio to the variability observed in all autosomes (52).

We used recommended parameters in Contammix (53) to estimate mitochondrial contamination rates and estimated contamination on the X-chromosome for all biologically male individuals using ANGSD (54). Mitochondrial and x-chromosomal contamination rates for all individuals sequenced were low (mitochondrial: < 2.5%; x-chromosomal: < 1 %, Dataset S2*A*). We estimated patterns of DNA damage using MapDamage 2 (55), observing high damage rates at the read termini ranging from 22% to 44%.

### Mitochondrial and y-Chromosomal DNA Analyses.

The mitochondrial haplotypes of the individuals were determined as described in Llamas et al., 2016. We excluded common indels and mutation hotspots at nucleotide positions 309.1C(C), 315.1C, AC indels at 515 to 522, 16182C, 16183C, 16193.1C(C), and C16519T. We embedded the consensus mitochondrial genomes in the existing mitochondrial tree (mtDNA tree Build 17 [18 Feb 2016]) using HaploGrep2 (56). To determine the Y-chromosomal haplogroups of the male individuals, we used yHaplo (57), identifying the most derived allele upstream and the most ancestral allele downstream in the phylogenetic tree of the International Society of Genetic Genealogy version 15.73 (July 11th, 2020; http://www.isogg.org/tree).

### Population Genetic Analyses.

We called variants for our 6 genomes on the targeted 1240 k SNP positions, with a read chosen at random to represent this position using pileupcaller (https://github.com/stschiff/sequenceTools), after trimming 8 bp from each end of the reads using bamUTIL (https://genome.sph.umich.edu/wiki/BamUtil) to reduce potential bias introduced by DNA damage. We merged the data with published genome-wide data from ancient (16–18, 58) and modern-day (15, 59, 60) South- and Central American individuals and a global set of high-coverage genome data from the SGDP (61).

We computed principal components of the present-day Central- and South American individuals restricting our analyses to the intersection of 597,503 SNPs between the 1,240 K SNP format and the HO dataset (HO merge) and projected all the ancient individuals, including the previously published ones, onto the first two components using the “lsqproject: YES”, and “shrinkmode: YES” options in smartpca (v.16680).

We used the qp3pop package in AdmixTools (62) to compute outgroup-f3-statistics with SEs computed with a weighted block jackknife over 5-Mb blocks. Analyses were performed using the 1,240 k SNP dataset. We used the inbreed: YES parameter to account for our random allele choice at each position. We generated a matrix of the outgroup-f3 values converted these to distances by taking the inverse of the values (17) and generated a neighbor-joining tree using MEGA X (63), choosing USA_USR1_AncientBeringian.SG (64) as the outgroup (Fig. 2).

We computed f4 statistics in AdmixTools (62) using the package qpDstat and f4mode: YES, printse: YES. To accommodate potential bias resulting from DNA damage, we calculated the statistics using both the whole data and a filtered dataset for which only 265,849 transversion substitution sites were retained.

### Kinship Analysis.

To investigate the degree of relationship using autosomal data, we employed READ (28) and BREADR (32), and calculated the pairwise mismatch rate (PMR) for the same genotype set using the tool pMMRCalculator (https://github.com/TCLamnidis/pMMRCalculator), as described in Mittnik et al. (30). We further employed a maximum likelihood approach based on genotype likelihoods implemented in the software lcMLkin (29), and a Hidden-Markov-Model-based approach to identify identity-by-descent fragments implemented in KIN (31). For all applications, we used standard parameters, and further details can be found in Supplementary Text.

We determined ROH using the software hapROH (https://github.com/hringbauer/hapROH) (33). We computed ROH using default parameters for two individuals with sufficient coverage: B1 and B4.

### Isotopic Analyses.

Bulk samples of tooth enamel for five individual teeth were sampled and mechanically cleaned and prepared for δ^13^C and δ^18^O light isotope ratios (ca. 20 mg) and ^20n^Pb/^204^Pb and ^87^Sr/^86^Sr heavy “radiogenic” isotope ratios (ca. 40 mg). One small tooth root fragment (ca. 40 mg) for B3 was used in lieu of tooth enamel for heavy isotope ratios only. Detailed methods are provided in Supporting Information (Dataset S3).

For light isotopes, pretreated samples were measured using a Kiel carbonate prep device and a Finnigan MAT 252 isotope ratio mass spectrometer. Data (Table 1 and Dataset S3) are reported in standard delta notation (δ) in parts per thousand, or per mil (‰) relative to Vienna Pee Dee Belemnite (VPDB), and analytical precision was >0.05‰ for δ^13^C and δ^18^O.

For heavy isotopes, ion chromatography was conducted in single aliquots using Pb-spec and Sr-spec resins. Strontium and lead isotope ratios (Table 1 and Dataset S3) were measured in separate runs on a Nu-Plasma multiple-collector inductively coupled plasma mass spectrometer using NBS-981 and NBS-987 standards, respectively (65).

### Radiocarbon Date Calibration and Modeling.

All calibration and modeling were done in OxCal 4.4 (66). Results are rounded by 10 y and italics denote results from Bayesian models. Since calibrated dates are nonnormal distributions, we report both medians (~) and 95% probability ranges. Terrestrial dates were calibrated with SHCal20 (67), the most appropriate curve for the research area (68). Along this part of the Pacific coast, atmospheric carbon was probably all from the Southern Hemisphere, since the Tropical Low-Pressure Belt does not currently bring any air from the Northern Hemisphere (69). Detailed descriptions of the Bayesian models, the calibration parameters, and the OxCal code can be found in *SI Appendix Chapter 1: Radiocarbon Date Calibration & Modeling*; the section “Lapses between Generations” and Contextual Relationships of *SI Appendix* 1 further explain the reasoning of model choices.

### Ethics Statement.

This study is fully authorized with permits from the Peruvian Ministry of Culture (Resolución Viceministerial No. 016-2018-VMPCIC/MC) and supported the Museo Cao, Complejo Arqueológico El Brujo, and the Fundación Wiese, Peru, with whom several of the authors have long-standing relationships. Study design, interim results, and final results have been shared in public talks in Peru and discussed with stakeholders, including the community of Magdelena de Cao. Our work was conducted in accordance with the wishes of these communities.

## Supplementary Material

Appendix 01 (PDF)

Dataset S01 (XLSX)

Dataset S02 (XLSX)

Dataset S03 (XLSX)

## Data Availability

All data needed to evaluate the conclusions in the paper are present in the paper and/or *SI Appendix*. Aligned sequencing reads for all individuals reported in this study are available from European Nucleotide Archive (ENA), Accession No: PRJEB81321 (70).

## Footnotes

^*^At the sites of Sipán, Lambayeque Valley; El Pueblo, Zaña Valley, San José de Moro, La Leche Valley; Dos Cabezas, Jequetepeque Valley; and Huaca de la Cruz, Virú Valley.
