# Supplementary material for: Family relations of Moche elite burials on the North Coast of Peru (~500 CE): Analyses of the Señora de Cao and relatives
Source: Proc Natl Acad Sci U S A. 2024 Dec 23;122(1):e2416321121. doi: 10.1073/pnas.2416321121 (PMC11725780; doi:10.1073/pnas.2416321121)
Supplement: Supplementary file 1 — Appendix 01 (PDF) [file pnas.2416321121.sapp.pdf]

Supporting Information for

Family Relations of Moche Elite Burials on the North Coast of Peru (~ 500 CE): Analyses of the Señora de Cao and Relatives

Jeffrey Quilter[A,*], Kelly Harkins[B], Régulo Fanco Jordan[C], Erik Marsh[D], Gabriel Prieto[E], John Verano[F], Steven LeBlanc[A], Nasreen Broomandkhoshbacht[B], John Krigbaum[E,*,1], Lars Fehren-Schmitz[B,G,*]

Corresponding Authors:
Jeffrey Quilter; Email: quilter@fas.harvard.edu
John Krigbaum; Email: krigbaum@ufl.edu
Lars Fehren-Schmitz; Email: lfehrens@ucsc.edu

This PDF file includes:

Supporting text
Figures S1 to S6
Legends for Datasets S1 to S3
SI References

Other supporting materials for this manuscript include the following:

Datasets S1 to S3

1. Radiocarbon Date Calibration & Modeling

Setting up the model

All modeling was done in OxCal 4.4 (1). Results are rounded by 10 years. Italics denote results from Bayesian models. Since calibrated dates are non-normal distributions, we report medians (~) and 95% probability ranges. Terrestrial dates were calibrated with SHCal20 (2). This is the most appropriate curve for this location (3). Along this part of the Pacific coast, it is likely that atmospheric carbon was exclusively from the Southern Hemisphere, since the Tropical Low-Pressure Belt does not currently bring any air from the Northern Hemisphere (4). Individual calibrations with SHCal20 are imprecise, even when measured errors are low, since most of the dates used in this paper have distributions that fall on a long plateau in carbon concentrations, about AD 440–520.

Calibrating human bones with marine diet inputs

Up to now, most radiocarbon dates from the site have been on terrestrial materials, because dates on human bones usually have very imprecise calibrations. This comes from the multiplying uncertainties of 1) the measured date, 2) each individual's marine protein diet percentage and 3) the marine reservoir effect (ΔR), since marine foods have different carbon concentrations than terrestrial ones.

ΔR is the difference between local and global surface marine radiocarbon, as estimated by Marine20 (5). ΔR is also a qualitative proxy for the degree of upwelling (6). Regional ΔR data can be queried in a continually updated database, http://calib.org/marine (7). Data on ΔR data in this region are mostly from recent centuries and ^{14}C ages are highly variable. For the latitude range 3–14°S, the database has 77 ^{14}C ages that can be used to estimate a ΔR and 74 are from a narrow span, with ^{14}C ages of 422–712 BP. Over this lapse, ΔR varies nearly three centuries, from -181 to 108 years. This variability is apparent even within shells that lived for around a single year, and is even more variable in six shells from contemporary Moche burials at Huaca de La Luna (6, 8). Hence for precise models, a weighted average of these dates may not be informative. Indeed, trial

runs showed that using shell-based ΔRs resulted in low agreement indices. Based on this, we took a different tack and allowed ΔR to float freely as an unknown uniform distribution. Each individual had a different estimate for marine diet, ranging from 30–71±18–24%, so each one had their own mixed calibration curve (see Isotopic Analysis, below). The large error ranges account for various sources of error, including the generalized baseline values, and this is reflected in the imprecise individual radiocarbon calibrations.

Estimating birth and death dates

To estimate birth and death dates, we used offsets based on the formation age of the dated tissue and the age at death, following the approach in Lane and Marsh (9), originally proposed by (10). For example, Señora de Cao (B3) was dated with a 1st molar, which erupted 6±1 years after birth. She died 25–30 years after her birth. The sample from B2's rib should be close to his death since rib bone tissue is more active in terms of bone turnover due to the continuous movement of the lungs. For the other three individuals, samples were taken from individual metatarsal bones that should approximate age of death for younger individuals. For the two adolescents B1s and B3s, we used an offset of 1±1 years; for the older B1, we used 5±3 years. This is adequate for our purposes, as it reflects the fact that the dated bone formed prior to death (11). All of these individuals died fairly young, so errors and offsets have a minor effect on the results and fall within our rounding of 10 years.

Lapses between generations

The close family relationships identified by genomic data are the principal constants in the chronological model. The main sequence of four generations begins with B4, generation 1. B4 likely did not have children before age 15 and lived to 25 at the most, so the model assumes a generation gap of 15–25 years before generation 2, which included B1 and B3's mother. She has not been identified by genetic data (and perhaps not buried here), but she has a place in the chronological model. She gave birth to B1 and B3 when she was 24±6 years old, the global historic trend (see details in Lane and Marsh 2023).

Finally, B1 fathered B1s between age 15 and his death, no later than age 25. Genomic data suggest B2 was either B4's son or grandson and the model accounts for this uncertainty by allowing a range of 15–60 years between their births. There is no clear data on determining familial relationships for B3s.

Contextual relationships

There are five architectural dates from building 2, which includes Tombs 1–3. One is a clear outlier (OxA-7007) (12). The other four do not have clear enough stratigraphic relationships to build a Bayesian model. For additional contextual details on the dates for each of the five buildings (also called Huacas), see Koons (13), Franco (14, 15), and Quilter et al. (12). We provide updated calibrations for all dates from the site (Dataset S1).

The date of Tombs 1–3 depends on the 11 terrestrial samples from this context. Three of these dates are unexpectedly early: the sacrificial rope from tomb 1 (Beta-208631), a textile wrapping from tomb 3 (Beta-212820), and the wood post from the entrance to the northwest patio (Beta-230126). Since there is no statistical overlap with the rest of the dates, we grouped them as a phase that pre-dates Tombs 1–3.

The remaining eight dates were grouped using Combine, post-calibration, assuming they were part of a single depositional event for Tombs 1–3. In this case, the depositional "event" assumes all deaths materials were harvested within a few years, within 10 years, the rounding precision we use here. Hence, this event should be less than a decade. The mat covering B3 was dated twice, so these dates were combined with R_Combine (Beta-230124 & UCIAMS-102538). The first of these, Beta-230124, was published with an error of ±40; the Beta report clarifies it is fact ±50 (Barkwill Love, personal communication). The two other dates from tomb 3 are on the cotton wrapping (Beta-212819) and the sacrificial rope around B3s's neck (Beta-208632). There are two previously unpublished dates on textiles (Amy Rodman, personal communication), but it is unclear which tomb they are from (CAMS-55919, CAMS-55921). There is a date from

the mat in tomb 2 (UCIAMS-102537) and a soil sample from tomb 1 that does not have clear context information (UCIAMS-102536). Next, we added the deaths of all five individuals in tombs 1–3: B1, B1s, B2, B3, and B3s, since the tomb was sealed and there is no evidence of later reopening. Finally, all of these events are modeled as being prior to a small hearth placed on the floor covering the tombs (Beta-230123).

Results

ΔR estimate

An unexpected result of the model is an independent ΔR estimate. Instead of using approximations based on shell dates, we let ΔR float freely, so the model provided an estimate based on the other constraints, namely precise generational gaps. Four of the dated individuals lived in the same decade or two; B4 lived two generations earlier. Based on this minor temporal difference, we assumed ΔR was the same for all five individuals, which converged at -270±72 years. This is significantly different from current data estimates from the region for later centuries, which comprise most of the available data (6, 8). However, it is consistent with ΔR estimates from 12 dates on shells found in Moche IV tombs 34 and 35 at Huaca de La Luna (6, 8). The five youngest shell dates have ^{14}C ages of 1701–1592±37–57 and ΔRs of -307 to -198±49–63 (but these are not updated for Marine20). Our estimate fits this trend and is from the same period, confirmed by two paired terrestrial dates from the same tombs (AA-82355 & AA-82354), which calibrate to ~AD 460 and 480 (340–580, 95%, combined range). We suggest that for now, our human-bone ΔR is more useful than shell-based ΔR for calibrating dates on human bone from this region and period. Our ΔR averages five individuals' mixed marine diets over 1–2 decades. In contrast, dates from shells that lived less than a year can vary so much it is difficult to identify a reliable ΔR average, which seems to reflect rapidly shifting vertical mixing in coastal waters. Our model's ΔR has a normal distribution, which echoes the normal distributions of the marine diet percentages. Future research could improve the ΔR with additional dates on human tissue, paired terrestrial dates, and more precise dietary estimates derived from a more robust dietary isoscape.

Tracking ΔR in time has environmental implications. For the decades leading up to the placement of Tombs 1–3, this paper's model and the shells from Huaca de La Luna independently agree on a strongly negative ΔR. This "may be explained by two possibly related mechanisms: (1) deepwater upwelling reduction generated by extended El Niño conditions, and/or (2) greater than modern El Niño frequency causing ^{14}C enrichment of surface water. Either or both of these mechanisms may have led to the torrential rains and flooding recorded in the archaeological record" (6).

Tombs 1–3 depositional event

The phase of unexpectedly early materials has three dates with similar medians, ~AD 340–360 on textile wrapping, rope, and a wood post, suggesting people interacted with other tombs from at least this time and actively reused old materials in new burials and constructions. The people who prepared the materials in these tombs probably made a conscious decision to re-use textiles that were made with materials harvested some 150 years prior, based on the medians. This suggests there were some five generations of sustained community interactions with burials, though we cannot say if these were carefully guarded heirlooms or simply looted from older tombs. Error ranges are wide for these dates. Their distributions do not have any meaningful overlap with that of the Tomb 1–3 depositional event, so they have a minimal impact on the model.

The date of this depositional event, ~AD 500 (440–540, 95%), depends on the overlapping distributions of 8 terrestrial samples and 5 death dates. This combination passes the chi-square test with an agreement index of 70%. Removing three dates from this group increases the agreement index markedly (UCIAMS-102536, Beta-212819, CAMS-55921). In two of these, contextual information is weak. However, we retain all dates since we have no a priori reason to remove them. Minor disagreements among dates could result from myriad sources such as reuse of older materials, field contamination, unclear context excavation or recording, different pretreatment protocols, or inter-laboratory differences. Excluding them has a negligible effect on the estimated date of the combined event.

This event also includes five estimated death dates for the individuals in tombs 1–3. Individually, their probability distributions are too imprecise to influence the date of the depositional event. Including them here narrows their error ranges substantially, for example, B3's calibrated death date has a 95% distribution spans six centuries, AD 280–850, but by including it in the burial event and cross-referencing the generational gap sequence, the 95% range is reduced to a single century. If we exclude B2's death from this event, it has no significant impact on his birth date, which is consistently modeled as around ~AD 480. This date makes it much more likely he is B4's grandson and B1 and B3's sibling, not their father. These results make it plausible that B1–B3 were siblings who died at the same time. Since B3s also died at this time, she was probably born within a few years of B1s. Her genomic data do not specify which family member she was, but her age makes it likely she was the daughter of B1, B2, or perhaps B3, since they were buried together, similar to B1 and his son B1s. Finally, the fire on the floor above the tombs took place much later, ~AD 650 (540–780, 95%) so it does not affect the model; a new sample associated with the sealing of the tomb may be able to improve the model.

Three architectural dates from the same building (2) and phase (D/F) have calibrated medians of ~AD 420–460 (250–600, 95%) (OxA-7005, OxA-7005, OxA-7008). Phase F is stratigraphically associated with Tombs 1–3 (12). The associated depositional events can provide clues to the building program leading up to the burial of Tombs 1–3. These constructions might have included the original burial of B4, who died at ~AD 460 (390–500, 95%). It is likely that phase D/F preceded the burial of Tombs 1–3, though probability tails overlap.

OxCal Code

```
Plot()
{
 Curve("SHCal20","shcal20.14c");
 R_Date("Charred material above Señora Coa tomb, Beta-230123",1420,50);
 Curve("Marine20","Marine20.14c");
 Delta_R("Unknown delta R",U(-500,200));
```

```
 Sequence("Before and after Tombs 1–3")
 {
  Boundary("Start older materials");
  KDE_Plot("Older materials")
  {
   Curve("=SHCal20");
   R_Date("Sacrificial rope, Señora group, Beta-208631",1750,40);
   R_Date("Threads from B3 textile wrapping, Beta-212820",1760,40);
   R_Date("Wood post, entrance to northwest patio, phases F/D, Beta-230126",1730,50);
  };
  Combine("Tombs 1–3")
  {
   //Deaths B1, B1s, B2, B3, B3s
   Mix_Curves("Mix B1","SHCal20","Unknown delta R",51,24);
   Date("Death B1",R_Date("Metatarsal B1",1670,15)+N(5,3));
   Mix_Curves("Mix B1s","SHCal20","Unknown delta R",37,24);
   Date("Death B1s",R_Date("Metatarsal B1s",1635,15)+N(1,1));
   Mix_Curves("Mix B2","SHCal20","Unknown delta R",71,20);
   R_Date("Death B2, rib",1725,20);
   Mix_Curves("Mix B3","SHCal20","Unknown delta R",45,23);
   Date("Death B3",R_Date("1st molar, B3",1660,15)-N(6,1)+U(25,30));
   Mix_Curves("Mix B3s","SHCal20","Unknown delta R",30,18);
   Date("Death B3s",R_Date("Metatarsal B3s",1655,15)+N(1,1));
   Curve("=SHCal20");
   //Removing three dates from this group increases the agreement index notably, but
even with them the agreement is fine: UCIAMS-102536, Beta-212819, CAMS-55921. The
combination passes the chi-square test in both cases. Also, it has a negligible effect on
the estimate date for the combined event.
   R_Date("JQ-2, tomb 1/05 soil, UCIAMS-102536",1645,15);
   R_Date("JQ-3, tomb 2, mat, UCIAMS-102537",1615,15);
   R_Combine("Tomb 3, mat")
   {
    R_Date("Petate (mat) covering mummy bundle. Beta-230124",1580,50);
    R_Date("JQ-4. Mat covering mummy bundle, UCIAMS-102538",1625,15);
   };
   R_Date("Tomb 3, sacrificial rope, Beta-208632",1580,40);
```

```
   R_Date("Tomb 3, wrapping, unprocessed cotton, Beta-212819",1550,40);
   R_Date("C. 062.18, unclear which tomb, CAMS-55919",1570,50);
   R_Date("C. 062.7, unclear which tomb, CAMS-55921",1550,40);
  };
  Boundary("=Charred material above Señora Coa tomb, Beta-230123");
 };
 //B3s has an unclear second-degree relationship with B3. We did not model this, since it would be a very wide range, and because B3s is already well constrained, since she died at the same time as B3.
 //Fathers' age at children's birth is assumed to be no younger than 15. The upper limit is their maximum estimated age of death.
 Mix_Curves("Mix B4","SHCal20","Unknown delta R",65,22);
 Date("Death B4",R_Date("Metatarsal B4",1745,15)+N(5,3));
 Date("Birth B3s",Date("=Death B3s")-U(12,15));
 Sequence("Grandfather B4–grandson B1–great-grandson B1s")
 {
  Boundary("Start family tree");
  Date("Birth B4",Date("=Death B4")-U(20,25));
  Interval("B4's age when B1 & B3's mother is born",U(15,25));
  Date("Birth, mother of B1 & B3");
  //Global average for mother's age at birth
  Interval("Mother's age when B1 is born",N(24,6));
  Date("Birth B1",Date("=Death B1")-U(25,30));
  Interval("Father's age when B1s is born",U(15,30));
  Date("Birth B1s",Date("=Death B1s")-U(12,13));
  Boundary("End family tree");
 };
 Sequence("Granddaughter B3")
 {
  Date("=Birth, mother of B1 & B3");
  Interval("Mother's age when B3 is born",N(24,6));
  Date("Birth B3",Date("=Death B3")-U(25,30));
 };
 Sequence("Grandfather B4–son or grandson B2?")
 {
  Date("=Birth B4");
```

```
  //This gap could be as little as 15 years, if B4 was 15 when he had B2 as a son. The
gap could be as much as 60 years, if B2 is his grandson: B4 had a daughter around his
death, age 25, and then she gave birth to B2 as a 35-year old.
  Interval("B4's age when B2 is born",U(15,60));
  Date("Birth B2",Date("=Death B2, rib")-U(20,25));
 };
};
```

2. Genetic Analyses

Preservation of the Human Remains

The human remains in this study were well-preserved due to the dry conditions of the coast of Peru, brick-lined tombs under additional layers of architecture, and textile and reed mat wrappings. Upon discovery, some individuals exhibited partially preserved soft tissue and hair (B1, B2), while others were skeletonized entirely (B1s, B3s, B4). For the individual known as the Señora de Cao's (B3), most of the skin was well preserved, even with tattoos still visible; however, the internal organs had disintegrated by the time of excavation. The better preservation of skin in the case of B3 might have been due to the preparation of her body after death, for example, washing the body in seawater and a coating of cinnabar, treatments that were not accorded to the other remains.

DNA Extraction and Sequencing Library Construction

The initial DNA extraction was performed following a silica-column-based protocol optimized for the recovery of small ancient DNA molecules, as described by Dabney and colleagues (16). Subsequently, we prepared double stranded (dsDNA) Uracil–DNA–glycosylase treated ("UDG-half") sequencing libraries using 25ul of DNA extract for each individual as described by Rohland et al. (17). After sequencing each library on a NextSeq500 sequencer (Illumina) for 2x75 cycles we observed only minimal DNA yield, with endogenous molecule content for all but one library (individual B1) being below 0.2% (Fig. S1). We subsequently modified our extraction protocol by adding a bleach treatment pre-digestion as described by Boessenkool et al. (18). 30mg of bone powder were first incubated in 1ml of 0.5% sodium hypochlorite solution (Sigma Aldrich) at

room temperature for 15 minutes. After centrifuging, the supernatant was discarded, and the remaining bone powder pellet washed using 1 ml molecular grade H_2O for three times. Extraction continued as described above. The new extracts were used to build UDG-half dsDNA libraries as described above, followed by the same sequencing approach. While the endogenous DNA yield for these libraries significantly increased (~150-600%) compared to the dsDNA-libraries without bleach treatment, it remained too low for sufficient genome-wide sequencing for most individuals (Fig. S1). To try retaining more DNA molecules we employed a new library protocol that uses directional splinted ligation of Illumina's P5 and P7 adapters to convert natively single-stranded DNA and heat denatured double-stranded DNA into sequencing libraries in a single enzymatic reaction (19). We followed the protocol as described by the authors, using 10µl of the bleach-treated DNA extract for each individual. For each individual, we made two ssDNA libraries, one strictly following the protocol of Kapp et al.(19) and one where we treated 10µl of DNA bleach-treated extract with UDG first by incubating it with 0.06 U/µl USER enzyme (NEB) and 1x Cutsmart Buffer (NEB) for 30 min at 37°C. The reaction was then inhibited by adding 0.12 U/µl UGI (NEB). All the UDG-half-treated extract was then used for the library construction. The UDG- (ssDNA_Bleach_UDG) and nonUDG (ssDNA_Bleach_noUDG) treated Libraries were then double barcoded in a PCR-reaction using primers containing sample-specific index sequence combinations as described in Kapp et al. (19) and sequenced on a NextSeq500 sequencer (Illumina) for 2x75 cycles. The ssDNA_Bleach_UDG libraries exhibit a slight increase in endogenous molecule yield compared to the two previously applied methods (dsDNA_noBleach_UDG; dsDNA_Bleach_UDG), however the highest endogenous DNA content for all individuals was observed for the ssDNA_Bleach_noUDG libraries (Fig. S1). Subsequently, the ssDNA_Bleach_noUDG libraries for each individual were selected for deep sequencing on several lanes of a HiSeq4000 (Illumina) sequencer for 2x150 cycles at Fulgent Genetics (Temple City, CA). exhausting the unique molecules preserved for most libraries and obtaining genome coverages ranging from 0.01x to 1.2x (Dataset S2a).

Kinship Analyses

To investigate the degree of relationship using autosomal data, we employed three pairwise mismatch rate (PMR) based tools, differing in the approaches to how PMR distribution is statistically evaluated and interpreted: READ (20), BREADR (21), and pMMRCalculator (https://github.com/TCLamnidis/pMMRCalculator). For all tools, we computed PMR using the randomly called 1240K SNP sites for the 6 individuals and four Moche commoners buried at El Brujo, reported previously (22), using default parameters. Both READ and BREADR produced consistent pairwise relatedness estimates for the individuals studied here (Fig. S3). We also calculated the pairwise mismatch rate (PMR) using the tool pMMRCalculator , as described in (23). The median PMR for pairs of individuals with more than 10,000 overlapping SNPs was determined to be 0.21188 (Dataset S2c). Pairings with lower PMR rates (0.142 to 0.195) likely represent more closely related individuals, with the lowest values being expected for first-degree relatives. The PMR rates and P0 values computed by READ are highly correlated (r2=0.9803; p= 0.0001). When plotting P0 against PMR we observe four clusters: the first includes the individuals determined to be 1st degree relatives by READ; the second includes the individuals determined as 2nd degree relatives by READ; with the pair B3-B2 that has only ~490 overlapping SNPs plotting between the first two clusters; the fourth cluster consists of unrelated individuals; the potential 3rd cluster, situated between the 2nd and the 4th has individuals that READ determined to be not 1st or 2nd degree relatives (the READ limit), but with PMR and P0 values (PMR: 0.188-0.194; P0: 0.194-0196) lower than the cluster of unrelated pairs, which could indicate a degree of relatedness higher than 2nd degree between the individuals (e.g., 3rd-5th degree)(20, 23).

We further employed a maximum likelihood approach based on genotype likelihoods implemented in the software lcMLkin (24). We called genotype likelihoods on variants observed at a minor allele frequency (MAF) of 5% in Native American individuals in the 1240k dataset for the six Huaca Cao Viejo individuals and 118 published ancient and modern-day Native American genomes (22, 25–29) using the SNPbam2vcf.py (https://github.com/COMBINE-lab/maximum-likelihood-relatedness-estimation/tree/master/src_python/SNPbam2vcf), and retaining total of 643,130 SNPs.

We choose a subset of individuals from different burial sites that, based on their geographic distribution and age, should not be related to determine the range of k0 (probability of two individuals not sharing an allele in a given site by IBD) and r (coefficient of relatedness). We observed a range of k0 = 0.932-0.997 and r= 0.034 – 0.003 when considering only pairs with over 10,000 SNPs overlapping, and a range of k0= 0.930-0.999 and r= 0.036-0.001 when including pairs with less than 10,000 SNPs. We used those ranges as a benchmark of r values among individuals that are definitely not directly biologically related, as suggested by Amorim et al.(30).

When plotting r against k0, as suggested in (23) we observe up to six distinct clusters of pairs that reflect different degrees of relationship (Fig. S4; Dataset S2f): first degree (parent-offspring), first degree (siblings), second degree, third degree, fourth degree, and a cluster that contains higher degrees of relatedness and unrelated individuals (24). We calculated the ratio of r to k0 which allows the determination of the boundaries for the observed clusters to assign degrees of relatedness to the individuals (23). Based on the previously observed k0 and r ranges for individuals unlikely to be related we can assume the r/k0 threshold for unrelated individuals to be at least 0.0365. The r/k0 thresholds are further defined as: 1st degree parent-offspring = ~1.1; 1st degree sibling ~0.55; 2nd degree relatives = 0.3 using the READ and PMR assignments as points of calibration. Due to the low number of 1st-degree relatives in the set of individuals, the thresholds come with significant uncertainty. The 1st and 2nd degree assignments of READ are in full agreement with the clusters defined using r and k0 from lcmlKIN. A fourth cluster with a threshold set at 0.13 correlates with individuals that were not assigned 1st or 2nd-degree relatives in READ and PMR, however, formed a cluster distinct from other unrelated individuals and most likely represents 3rd-degree related pairs (expected k=0.125). There is a potential 5th cluster with a threshold around 0.06 that could represent 4th or higher degree relatives (24), however, due to the overall low number of available non-admixed American genomes to calculate genotype likelihoods, and the lack of known relatives of higher degrees in this group, it is impossible to determine empirically supported r/k0 thresholds. Thus, we caution that any threshold for a higher

degree of relatedness discussed here is rather arbitrary or comes with statistical uncertainty.

The fifth tool we used to determine relatedness, KIN, employs a hidden Markov model (HMM)-based approach to estimate genetic kinship and IBD. Compared to lcmlKIN, the tool is more efficient in dealing with the characteristics of very low-coverage ancient genomes (31). The method can detect up to 3rd-degree relatives and differentiates between siblings and parent-child relationships. We used their default parameters on a set of BAM files including the 6 patio burial individuals, and four unrelated commoner individuals from the broader El Brujo complex. Observations made with KIN largely correlate with those made with lcmlKIN (Dataset S2d)

We determined Runs of Homozygosity (ROH) using the software hapROH (https://github.com/hringbauer/hapROH) (32) which has been successfully tested to produce reliable results for pseudo-haploid genotype data from a targeted set of 1.24 million single nucleotide polymorphisms widely used in ancient DNA, here referred to as 1240k (32, 33). We computed ROH using default parameters for two individuals with sufficient coverage: B1 and B4. The frequency of long ROH (sum_ROH > 20 cM = 54.45; Fig S5) observed for B4 indicates an increased degree of parental relatedness, most likely being second cousins (5th degree). We observed no long ROH for B1.

Family Tree reconstruction

We reconstructed the family tree for the Huaca Cao Viejo individuals drawing on several lines of evidence: the pairwise relatedness estimates obtained with the methods described above; the mitochondrial haplotypes to indicate maternal kinship; the individual age at death, excluding sub-adult individuals as potential parents; the radiocarbon dates obtained from skeletal remains of the individuals, and other information deriving from the archaeological context. The relatedness estimates obtained with the five previously described methods were largely consistent (Dataset S2c) and allowed us to produce a plausible pedigree spanning at least four generations.

We started reconstructing the core family tree with the individuals for which we obtained the most data, and for which relatedness estimates were highly statistically supported. We used PRIMUS (34), which uses genome-wide estimates of pairwise IBD to identify families and quickly reconstruct pedigree trees, to calculate the most likely family tree models fitting the genetic data. For PRIMUS we used the IBD estimates obtained from KIN, as well as the mitochondrial haplotype, and age and sex information. We continued eliminating unlikely models based on the contextual archeological data. This way yielded one feasible family tree (Fig. 4), which correlates with the manual reconstruction of the family tree described in the following.

The earliest dated individual of the burial group is B4, the young adult biological male. The lack of grave goods associated with this burial, and the fact that the individual was poorly preserved suggests that this might be a secondary burial. B4 is a 2nd degree relative to individuals B1 and B3 (Señora de Cao), both determined to be 1st degree relatives. The ratio of r to k0 obtained with lcmlKIN indicates that B1 and B3 should be siblings (r/k0 = 1.7239), which is supported by the KIN estimates, which means that B4 must be their grandfather. B4 does not share the mitochondrial lineage with the latter two individuals, which supports that assumption. The analyses further suggest that B1 and B1s are 1st-degree relatives of the parent-offspring type (Dataset S2c; r/k0 = 0.8787). B1s is a subadult biological male individual of pre-reproductive age that was buried as a likely sacrifice with B1, which suggests that the latter must have been B1s's father. This position in the family tree is further supported by the observation that B3 and B1s are 2nd-degree relatives (aunt-nephew) and that B4 and B1s are 3rd-degree relatives (Dataset S2c). It must be mentioned that B3 and B1s only have ~6000 SNPs overlap. However, all methods utilized support 2nd degree relatedness with a degree of certainty (e.g., READ Z_upper=1.9602; Z_lower=-2.2622). B1s exhibits the same mitochondrial haplotype like their father B1 and aunt B3, which indicates that B1s's mother must have exhibited some degree of matrilineal relatedness to the mother of B1 & B3 (their grandmother). The frequency of long ROH observed in the genome of B4 indicates that the parents of this individual must have been 5th-degree relatives (e.g., second cousins). While we have no direct evidence, we suggest that if 2nd cousin marriages were common / accepted in

high-status Moche families, it could also explain the observed matrilineal relatedness between the mother of B1s and the mother of B1 & B3.

All methods also give weak, but consistent support for a 2nd degree relatedness (e.g., grandparent-grandchild, aunt-niece) between B3 (La Señora) and the subadult biological female co-burial B3s. However, the number of SNPs overlapping between both individuals is very low (~1,800 SNPs), resulting in a non-significant upper-Z score in the READ analysis, while the lower-Z score is significant, meaning they could definitely not be 1st-degree relatives but less than 2nd-degree relatives. The SNP overlap between B3s and the assumed sibling of B3, B1, is also very low (3,600-5,000 SNPs depending on method), but both lcmlKin and the clustering behavior when plotting P0 against PMR indicate that the pair could be 2nd or 3rd-degree relatives. B3 was probably not older than ~30 years when they died, which would make it less likely that a grandchild (B3s was 12-15 years at death, Dataset S1a) would have been buried simultaneously with the Señora de Cao. There is no archaeological evidence that the tomb might have been reopened to bury B3s long after B3. Considering the low SNP overlap between the pairs B3-B3s, and B1-B3s we cannot exclude that the relatedness estimates are biased. The individual could have been the child of a sibling of B1 and B3. We suggest that B3s was to some degree biologically related to B3 and other individuals of the family and reflect this uncertainty in the family tree (Fig. 4).

We only obtained very low coverage for the biologically male individual buried in Tomb 2 (B2). The genetic data indicate that the individual shares the mitochondrial D haplotype with B1, B1s, and B3 (mtD1). Even though the genetic overlap between B2 and the other individuals is minimal, READ still indicates that the individual is a 1st degree relative to B1 with some degree of certainty (~1,300 SNPs overlap, Z_upper = 1.9635, Z_lower = -1.7813), which is supported by both KIN and lcmlKIN. However, the methods are not consistent with regards to the type of first-degree relationship they suggest: KIN indicates a sibling relationship (Log Likelihood = 4.7), while lcmlKIN hints at a parent-offspring relationship (r/k0 = 6.51). When comparing B4 to B2 lcmlkin and KIN indicate that they could be 2nd degree relatives, however, again the SNP overlap is too low (1459 SNPs).

The PMR for the pair is 0.1803, which falls between the ranges determined for 2nd and 3rd degree relatives. The SNP overlap between B2 and any other individual of the group is <1,000 SNPs, precluding the ability to determine relatedness. Based on our observations, we are certain that B2 is biologically related to the other Huaca Cao Viejo individuals; however, we cannot determine the exact degree that would be necessary to fit the individual into the family tree–both our manual approach and the automated approach using PRIMUS indicate that the most likely model would be that B2 is either a sibling of both B1 and B3, or the parent of both. We indicate both possibilities in our reconstructed family tree, though discuss that the data calibrations and archaeological context favor a model that assumes B2 to be a sibling of B1 and B2 (Fig. 4).

3. Isotopic Analysis

Isotope analysis was conducted at the University of Florida in the Bone Chemistry Lab, Department of Anthropology and in the IRMS and ICP-MS laboratories in the Department of Geological Sciences. Samples analyzed included tooth crowns with tooth enamel, except for La Senora (B3) that included only a portion of tooth root (~50 mg). Sampled tooth enamel ‘chunks’ were mechanically abraded under stereomicroscopy to remove adhering dentine and exogenous materials using an NSK dental drill and mounted handpiece outfitted with a Brassler tungsten-carbide tapered drill bit. Cleaned tooth chunks were inspected under the microscope and parsed into two groups for light isotope ($\delta^{13}C$ and $\delta^{18}O$) and heavy isotope ($^{87}Sr/^{86}Sr$ and $^{20n}Pb/^{204}Pb$) analysis.

For each sample, the smaller tooth chunk (ca. 20 mg) was ground using an acid-cleaned agate set and sample powder weighed and loaded into a 1.5 mL microcentrifuge tube and oxidized using 2.5% sodium hypochlorite (NaOCL) for 8 hours to remove exogenous organic material. Samples were then centrifuged and rinsed to neutral pH using Milli-Q H_2O and then pretreated with 0.2 M acetic acid (CH_3COOH) for 8 hours. Samples were lyophilized for 48 hours, and then loaded for IRMS. Samples were reacted with 100% phosphoric acid at 70 °C and reaction time was 10 minutes.

Cleaned tooth enamel (and tooth root) samples were processed for heavy isotopes in the Clean Lab. Samples were placed in pre-cleaned Teflon vials, weighed and dissolved in 8

N nitric acid (HNO_3) (optima) and capped at 100 °C (overnight). Samples were subsequently dried on the hot plate and dried residues prepared for trace element analysis by adding 0.8 N HNO_3 spiked with 8 ppb Re and Rh to each weighed sample, aiming for ~2000x final dilution. Trace elements and rare earth elements were measured using an Element 2 HR-ICP-MS in medium resolution with Re and Rh internal standards, following the procedure outlined in Kamenov et al. (35). Remaining samples were subsequently processed for lead (Pb) and strontium (Sr) in single aliquots through ion chromatography using Dowex® 1X8, 100-200 mesh, ion-exchange resin for strontium and lead, respectively (Sr-spec and Pb-spec, Eichrom Technologies, Inc.). Strontium results were measured relative to NBS 987 ($^{87}Sr/^{86}Sr$=0.710246 ± 0.000030). Pb values were measured using Tl normalization and reported relative to NBS 981 ($^{206}Pb/^{204}Pb$=16.937 ± 0.004, $^{207}Pb/^{204}Pb$=15.490 ± 0.003, and $^{208}Pb/^{204}Pb$=36.695 ± 0.009).

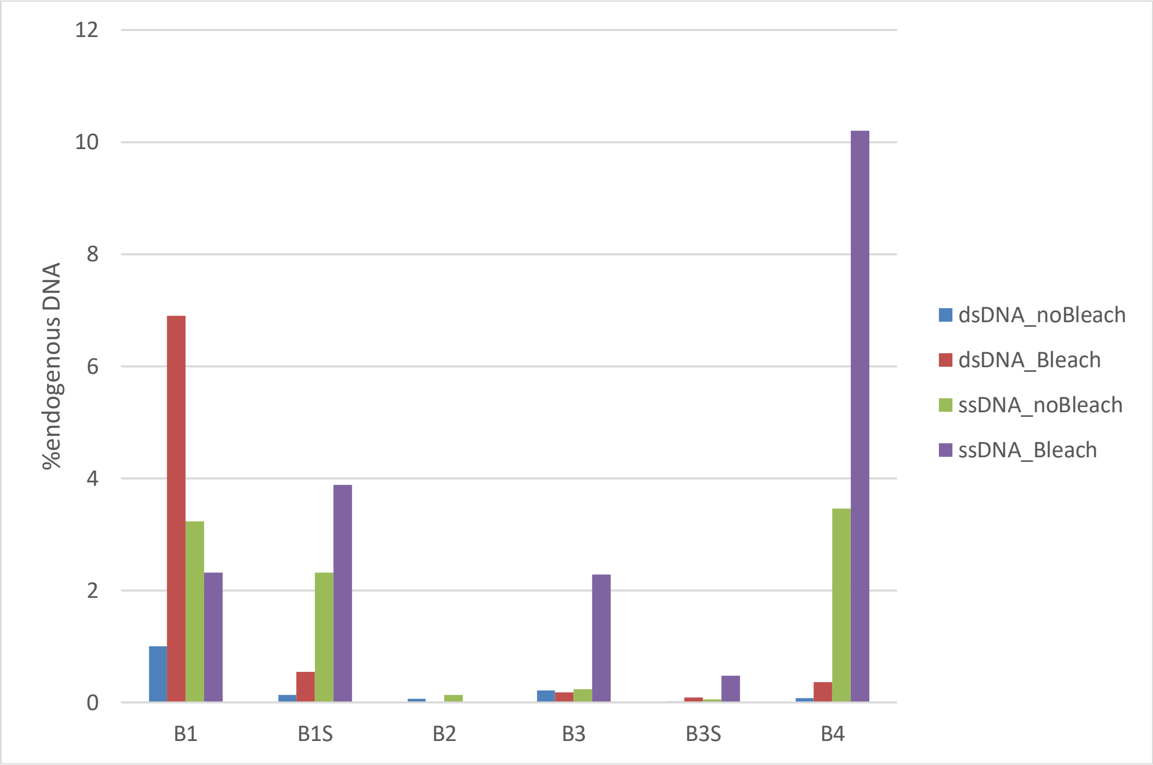

Fig. S1. Endogenous DNA yields for the different combined extraction and library protocols.

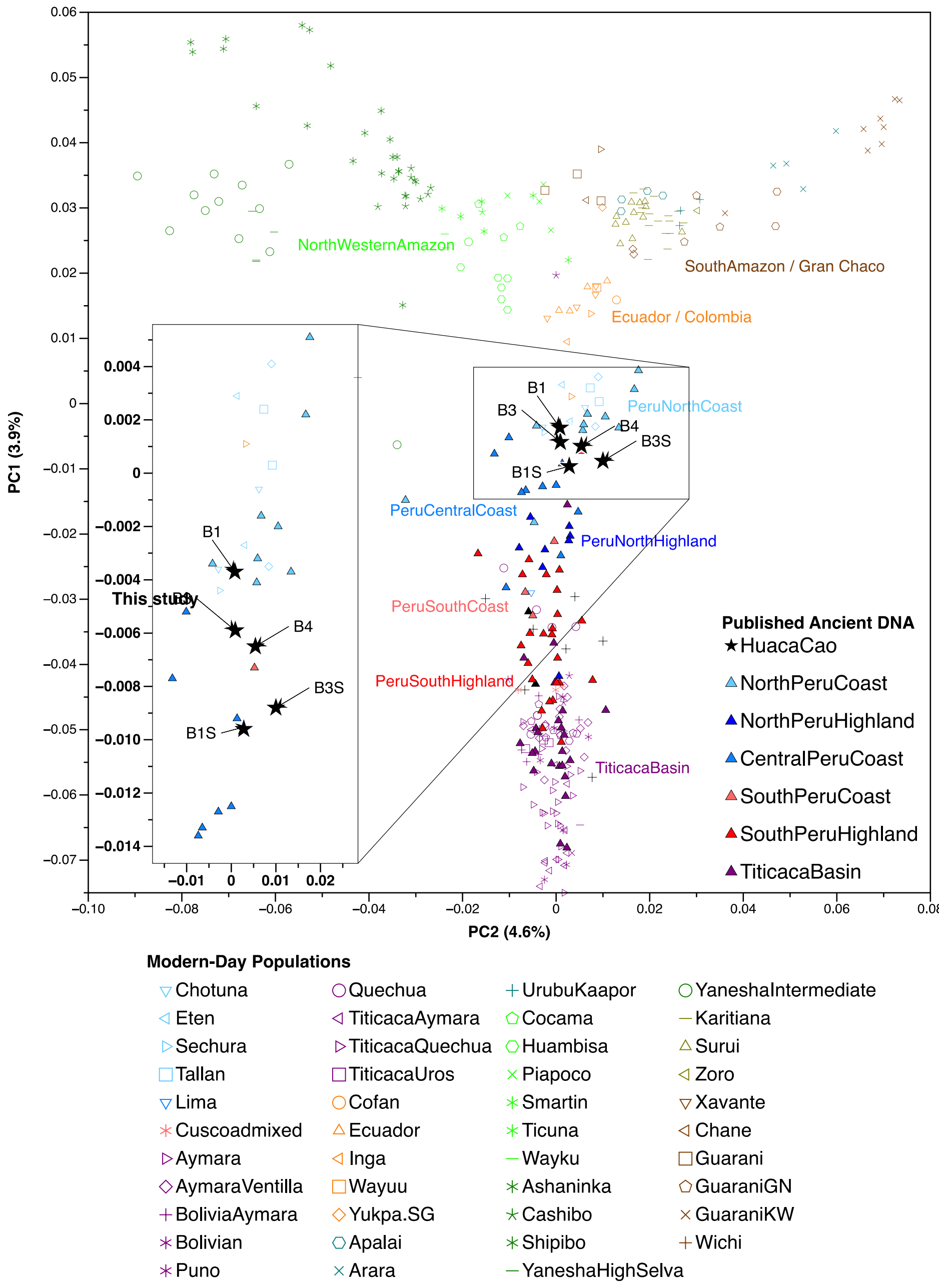

Fig. S2. Extended version of the PCA Plot (Figure 2A), including the population names of the modern-day populations used to compute the PCA.

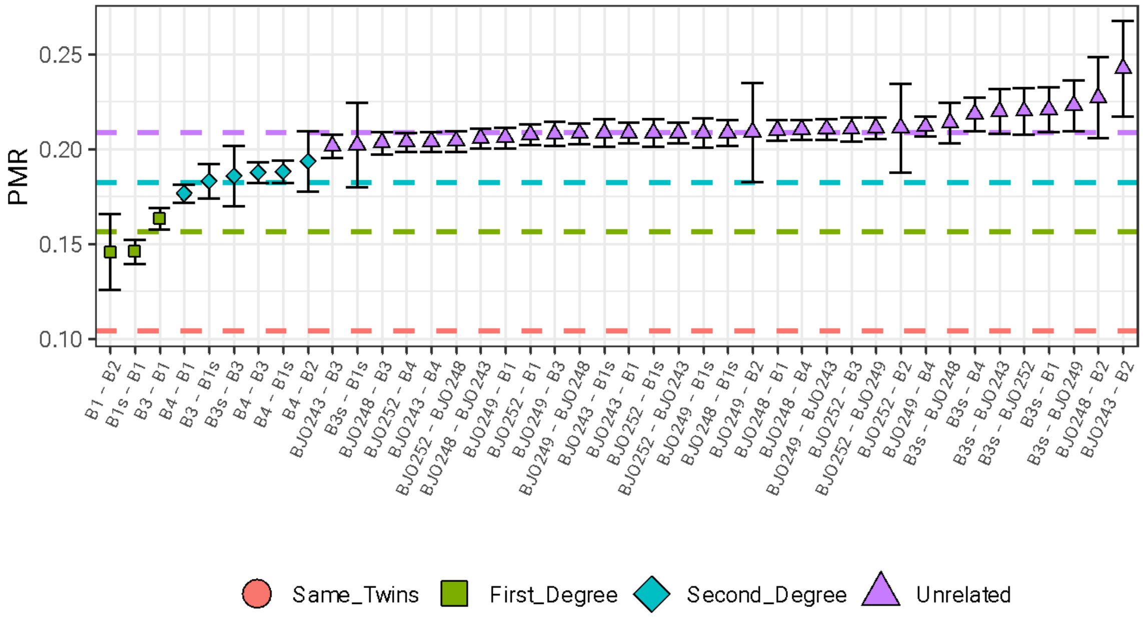

Fig. S3. Plot showing the genetic relatedness between paired Huaca Cao individuals, and the unrelated El Brujo reference individuals as determined by BREADR.

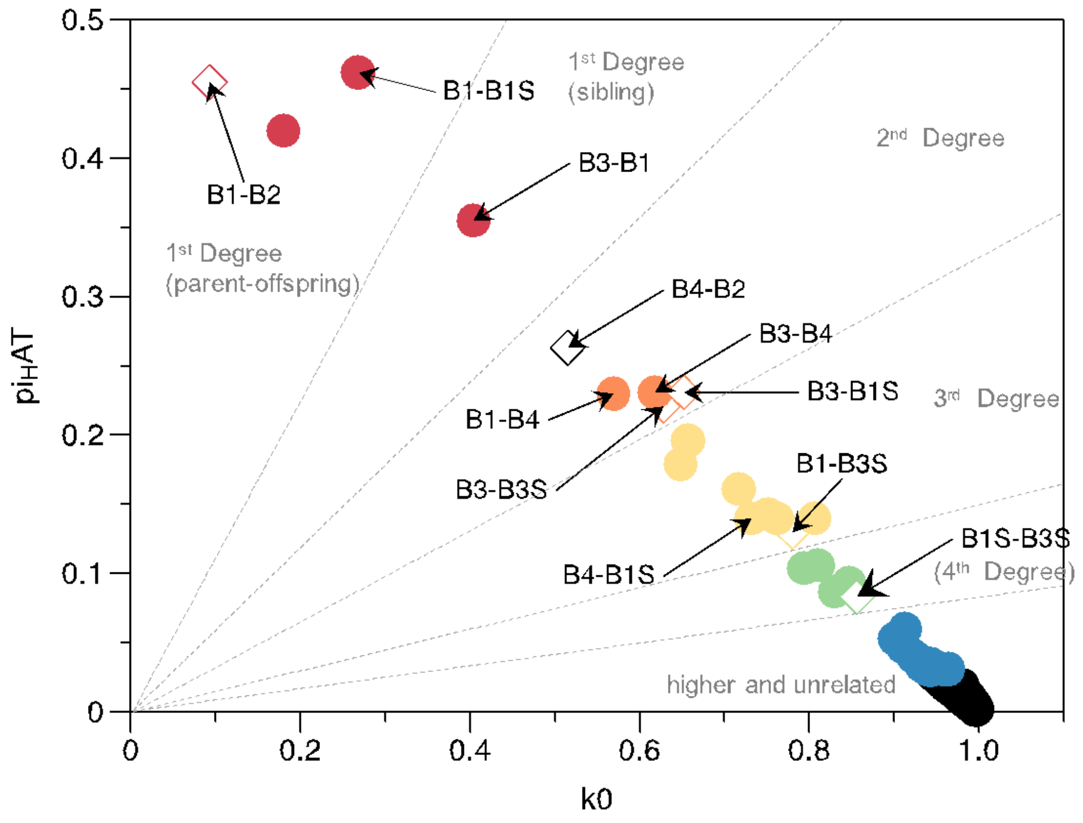

Fig. S4. k0 as estimated with lcmlKIN plotted against coefficient of relatedness r. Clusters of different degrees of relatedness emerge when plotting these measures. Unfilled symbols show pairwise comparisons between individuals with less than 5k SNPs overlap.

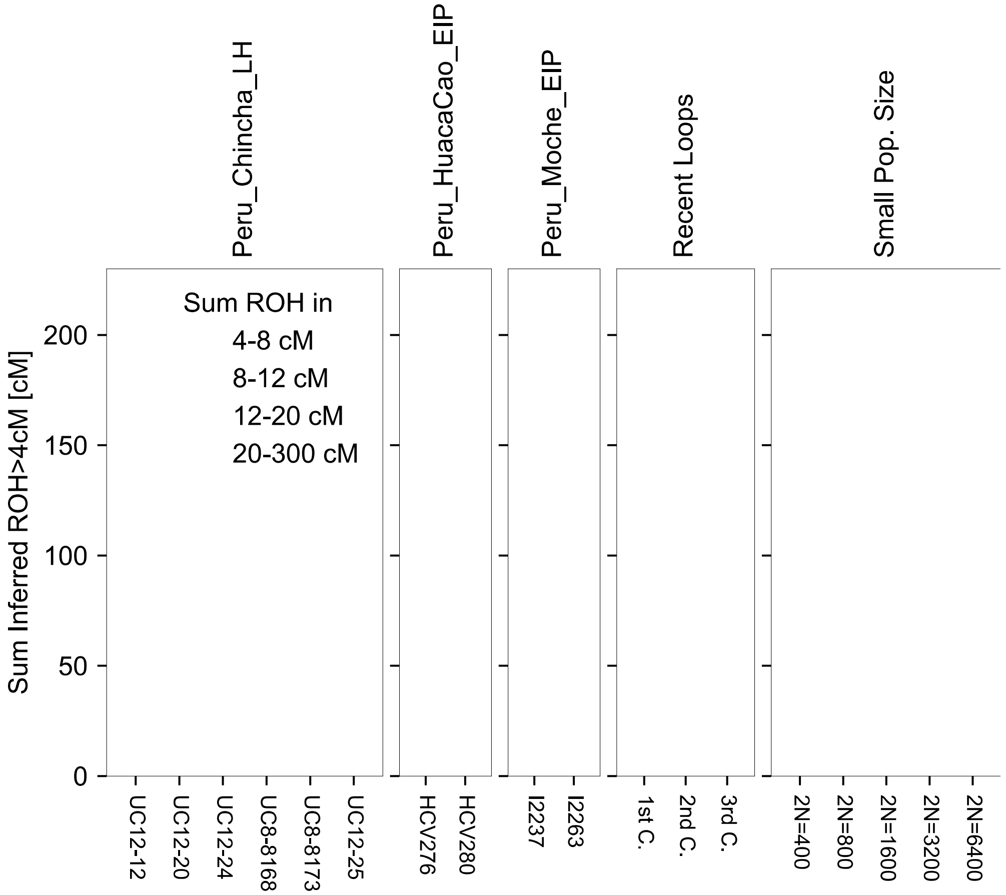

Fig. S5. ROH estimates for B1 (HCV280) and B4 (HCV276), and ancient reference genomes from Chincha (25), and individuals buried at El Brujo (25). Each individual is represented by stacked vertical bars, where the length of each bar is determined by the ROH of this individual falling into four length classes (4–8, 8–12, 12–20, and >20 cM, color-coded).

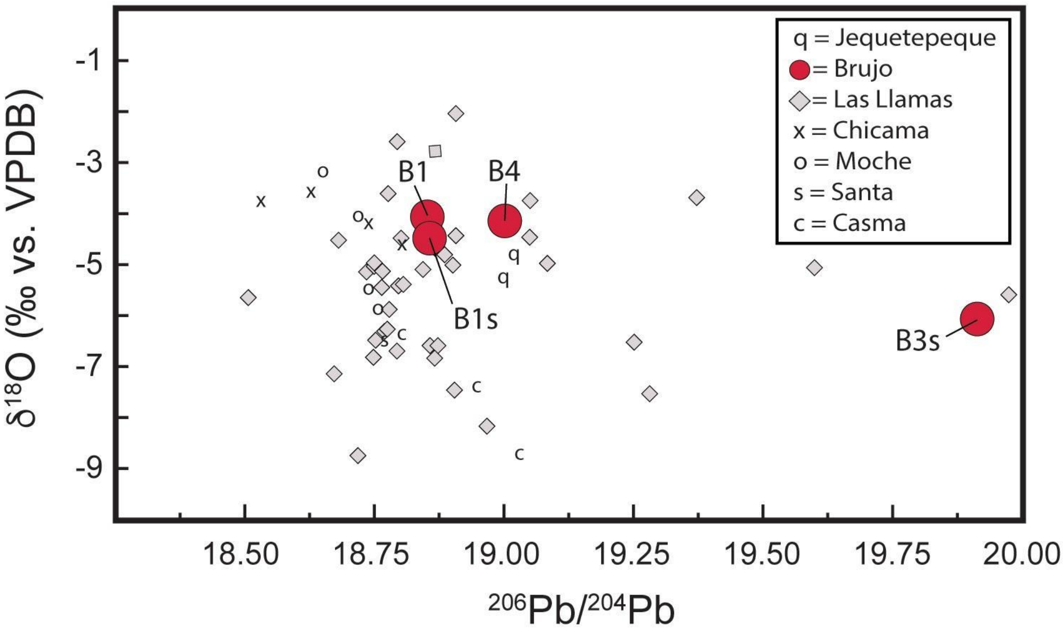

Fig. S6. Select lead ($^{206}Pb/^{204}Pb$) and oxygen ($\delta^{18}O$) isotope values for Huaca Cao individual tooth enamel and comparative site samples (Table S4), including nearby Huanchaquito-Las Llamas (Dataset S3). Señora de Cao (B3) not included, because only root dentin assayed for lead (and strontium), but not oxygen (or carbon).

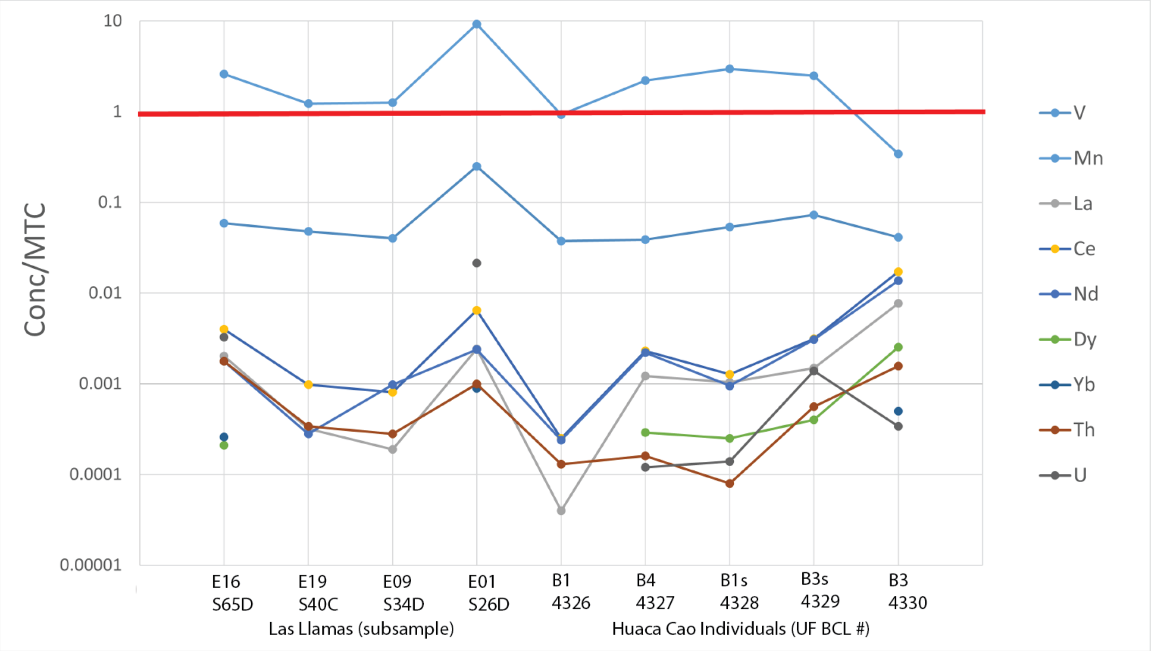

Fig. S7. Maximum Threshold Concentration (MTC) data for select human tooth enamel samples from Huanchaquito -- Las Llamas (subsample) and Huaca Cao individuals included in this study. B3 (La Senora) is tooth root, but included for comparison with tooth enamel. MTC values >1 suggest diagenetic alteration (Kamenov et al., 2018).

Dataset S1 (separate file). Radiocarbon Date Calibrations.

Dataset S2 (separate file). Genetic Results including sequencing statistics, relatedness estimates using the different computational tools, and f-statistics.

Dataset S3 (separate file). Isotope Results including concentrations and quality statistics for different investigated isotope systems

SI References

1. C. Bronk Ramsey, Bayesian Analysis of Radiocarbon Dates. *Radiocarbon* **51**, 337–360 (2009).

2. A. G. Hogg, *et al.*, SHCal20 Southern Hemisphere Calibration, 0–55,000 Years cal BP. *Radiocarbon* **62**, 759–778 (2020).

3. E. J. Marsh, *et al.*, IntCal, SHCal, or a Mixed Curve? Choosing a 14C Calibration Curve for Archaeological and Paleoenvironmental Records from Tropical South America. *Radiocarbon* **60**, 925–940 (2018).

4. S. Ancapichún, J. Pawlyta, A. Z. Rakowski, D. Sieczkowska, Influence of Air Parcels from Northern and Southern Hemispheres on Radiocarbon-Based Inca Chronology. *Radiocarbon* **64**, 1431–1446 (2022).

5. T. J. Heaton, *et al.*, Marine20—The Marine Radiocarbon Age Calibration Curve (0–55,000 cal BP). *Radiocarbon* **62**, 779–820 (2020).

6. M. F. Etayo-Cadavid, *et al.*, Marine radiocarbon reservoir age variation in Donax obesulus shells from northern Peru: Late Holocene evidence for extended El Niño. *Geology* **41**, 599–602 (2013).

7. P. J. Reimer, R. W. Reimer, A Marine Reservoir Correction Database and On-Line Interface. *Radiocarbon* **43**, 461–463 (2001).

8. M. F. Etayo-Cadavid, C. F. T. Andrus, K. B. Jones, G. W. L. Hodgins, Subseasonal variations in marine reservoir age from pre-bomb *Donax obesulus* and *Protothaca asperrima* shell carbonate. *Chemical Geology* **526**, 110–116 (2019).

9. K. Lane, E. J. Marsh, *Absolute Chronology* revisited: Integrating precise Bayesian models from Machu Picchu with Inca ethnohistoric praise narratives. *Quaternary International* (2023). https://doi.org/10.1016/j.quaint.2023.11.006.

10. A. R. Millard, *et al.*, Scottish soldiers from the Battle of Dunbar 1650: A prosopographical approach to a skeletal assemblage. *PLOS ONE* **15**, e0243369 (2020).

11. D. H. Ubelaker, Radiocarbon analysis of human remains: a review of forensic applications. *J Forensic Sci* **59**, 1466–1472 (2014).

12. J. Quilter, *et al.*, The Well and The Huaca: Ceremony, Chronology, and Culture Change at Huaca Cao Viejo, Chicama Valley, Peru. *Andean Past* **10**, 101–132 (2012).

13. J. Quilter, M. L. Koons, The Fall of the Moche: A Critique of Claims for South America's First State. *Latin American Antiquity* **23**, 127–143 (2012).

14. R. Franco, “El Complejo Arqueológico el Brujo: Cronología y Secuencia Cultural” in *El Brujo: Huaca Cao, Centro Ceremonial Moche En El Valle de Chicama*, B. E. Mujica, E. H. Maio, Eds. (Fundación Wiese, 2021), pp. 22–35.

15. R. Franco, “Excavationes en el Recinto Ceremonial de la Equina Superior Noreste de la Huaca Cao Viejo” in *El Brujo: Huaca Cao, Centro Ceremonial Moche En El Valle de Chicama*, B. E. Mujica, E. H. Maio, Eds. (Fundación Wiese, 2021), pp. 52–83.

16. J. Dabney, *et al.*, Complete mitochondrial genome sequence of a Middle Pleistocene cave bear reconstructed from ultrashort DNA fragments. *Proceedings of the National Academy of Sciences of the United States of America* **110**, 15758–63 (2013).

17. N. Rohland, E. Harney, S. Mallick, S. Nordenfelt, D. Reich, Partial UDG-treatment for screening of ancient DNA. *Philos.Trans.R.Soc.Lond B Biol.Sci.* (2014).

18. S. Boessenkool, *et al.*, Combining bleach and mild pre-digestion improves ancient DNA recovery from bones. *Molecular Ecology Resources* (2016). https://doi.org/10.1111/1755-0998.12623.

19. J. D. Kapp, R. E. Green, B. Shapiro, A Fast and Efficient Single-stranded Genomic Library Preparation Method Optimized for Ancient DNA. *Journal of Heredity* **112**, 241–249 (2021).

20. J. M. M. Kuhn, M. Jakobsson, T. Günther, Estimating genetic kin relationships in prehistoric populations. *PLOS ONE* **13**, e0195491 (2018).

21. A. B. Rohrlach, J. Tuke, D. Popli, W. Haak, BREADR: An R Package for the Bayesian Estimation of Genetic Relatedness from Low-coverage Genotype Data. [Preprint] (2023). Available at: https://www.biorxiv.org/content/10.1101/2023.04.17.537144v1 [Accessed 10 May 2023].

22. N. Nakatsuka, *et al.*, A Paleogenomic Reconstruction of the Deep Population History of the Andes. *Cell* **181**, 1131-1145.e21 (2020).

23. A. Mittnik, *et al.*, Kinship-based social inequality in Bronze Age Europe. *Science* (2019).

24. M. Lipatov, K. Sanjeev, R. Patro, K. R. Veeramah, Maximum Likelihood Estimation of Biological Relatedness from Low Coverage Sequencing Data. *bioRxiv* 023374 (2015). https://doi.org/10.1101/023374.

25. J. L. Bongers, *et al.*, Integration of ancient DNA with transdisciplinary dataset finds strong support for Inca resettlement in the south Peruvian coast. *PNAS* **117**, 18359–18368 (2020).

26. J. V. Moreno-Mayar, *et al.*, Early human dispersals within the Americas. *Science* **362**, eaav2621 (2018).

27. C. Posth, *et al.*, Reconstructing the Deep Population History of Central and South America. *Cell* **175**, 1185-1197.e22 (2018).

28. M. Raghavan, *et al.*, Genomic evidence for the Pleistocene and recent population history of Native Americans. *Science* **349** (2015).

29. M. Rasmussen, *et al.*, Ancient human genome sequence of an extinct Palaeo-Eskimo. *Nature* **463**, 757–62 (2010).

30. C. E. G. Amorim, *et al.*, Understanding 6th-century barbarian social organization and migration through paleogenomics. *Nature Communications* **9**, 3547 (2018).

31. D. Popli, S. Peyrégne, B. M. Peter, KIN: a method to infer relatedness from low-coverage ancient DNA. *Genome Biology* **24**, 10 (2023).

32. H. Ringbauer, J. Novembre, M. Steinrücken, Parental relatedness through time revealed by runs of homozygosity in ancient DNA. *Nat Commun* **12**, 5425 (2021).

33. H. Ringbauer, M. Steinrücken, L. Fehren-Schmitz, D. Reich, Increased rate of close-kin unions in the central Andes in the half millennium before European contact. *Curr Biol* **30**, R980–R981 (2020).

34. J. Staples, *et al.*, PRIMUS: Rapid Reconstruction of Pedigrees from Genome-wide Estimates of Identity by Descent. *The American Journal of Human Genetics* **95**, 553–564 (2014).

35. G. D. Kamenov, E. M. Lofaro, G. Goad, J. Krigbaum, Trace elements in modern and archaeological human teeth: Implications for human metal exposure and enamel diagenetic changes. *Journal of Archaeological Science* **99**, 27–34 (2018).
